# Challenges and advances of organic electrode materials for sustainable secondary batteries

**DOI:** 10.1002/EXP.20220066

**Published:** 2022-07-27

**Authors:** Ruijuan Shi, Shilong Jiao, Qianqian Yue, Guangqin Gu, Kai Zhang, Yong Zhao

**Affiliations:** ^1^ School of Materials, Key Lab for Special Functional Materials of Ministry of Education Henan University Kaifeng China; ^2^ Frontiers Science Center for New Organic Matter Renewable Energy Conversion and Storage Center (RECAST) Key Laboratory of Advanced Energy Materials Chemistry (Ministry of Education) College of Chemistry Nankai University Tianjin China; ^3^ Haihe Laboratory of Sustainable Chemical Transformations Tianjin China

**Keywords:** advanced strategies, challenges, characterization techniques, organic electrode materials, redox reaction mechanism

## Abstract

Organic electrode materials (OEMs) emerge as one of the most promising candidates for the next‐generation rechargeable batteries, mainly owing to their advantages of bountiful resources, high theoretical capacity, structural designability, and sustainability. However, OEMs usually suffer from poor electronic conductivity and unsatisfied stability in common organic electrolytes, ultimately leading to their deteriorating output capacity and inferior rate capability. Making clear of the issues from microscale to macroscale level is of great importance for the exploration of novel OEMs. Herein, the challenges and advanced strategies to boost the electrochemical performance of redox‐active OEMs for sustainable secondary batteries are systematically summarized. Particularly, the characterization technologies and computational methods to elucidate the complex redox reaction mechanisms and confirm the organic radical intermediates of OEMs have been introduced. Moreover, the structural design of OEMs‐based full cells and the outlook for OEMs are further presented. This review will shed light on the in‐depth understanding and development of OEMs for sustainable secondary batteries.

## Graphical abstract

Organic electrode materials with merits of bountiful resources, structural designability, and sustainability offer an attractive solution to develop the degradable and eco‐friendly batteries. This review is focusing on the issues and complex redox mechanism faced by organic electrode materials from microscale to macroscale level, in combination with the state‐of‐art strategies and in‐depth outlook, promoting sustainable development and non‐hazardous disposal of future organic batteries.

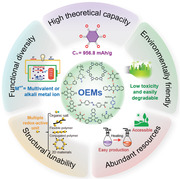

## 1 INTRODUCTION

**FIGURE 1:**
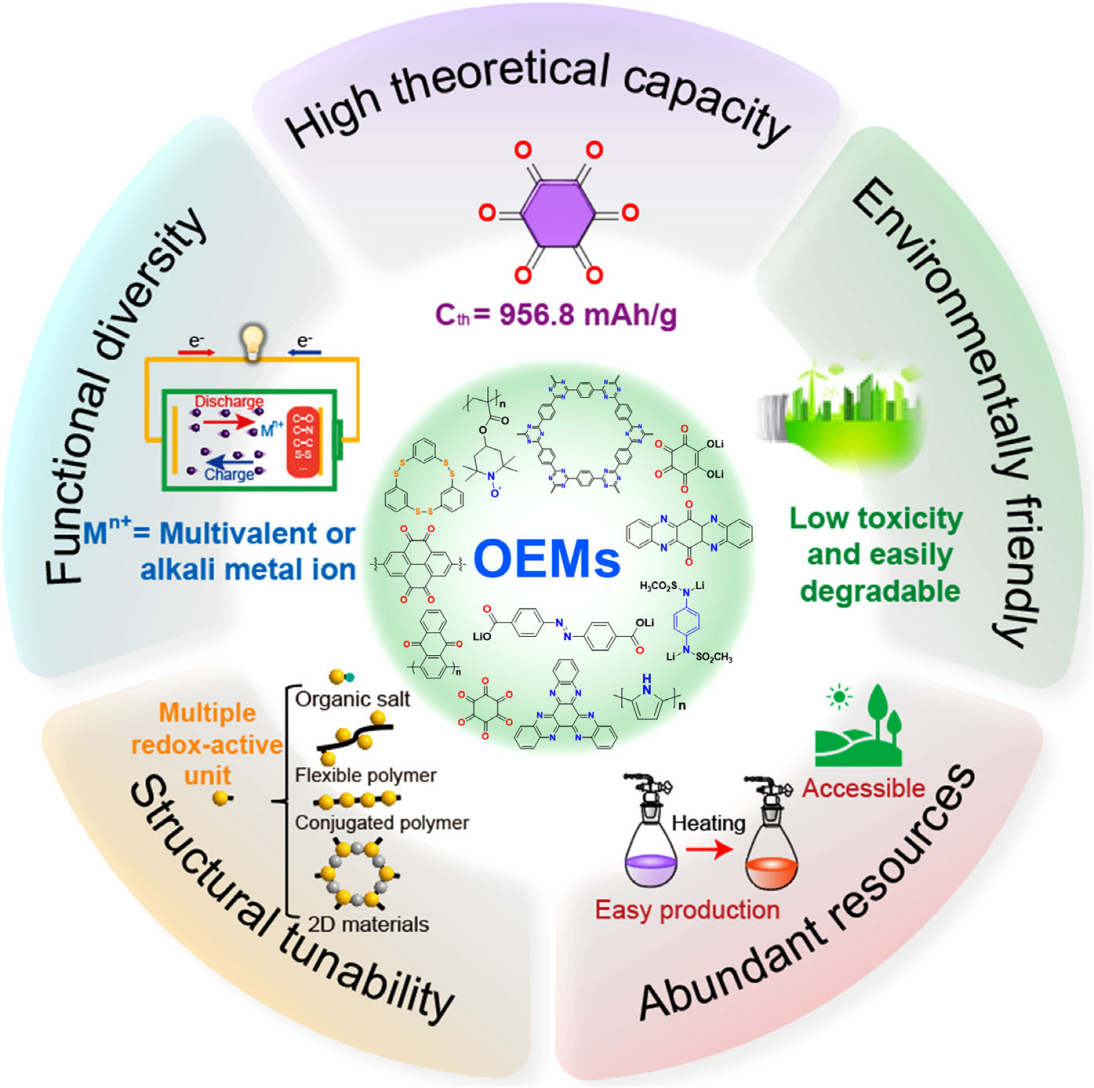
Advantages of OEMs in rechargeable secondary batteries

The rising development of new energy electric vehicles, large‐scale fixed energy storage, and the national smart grid has put forward high requirements on the mass energy density, cycle life, and resource reserves of energy storage devices.^[^ ^1^, ^2^, ^3^, ^4^ ^]^ Traditional lithium ion batteries (LIBs) with limited theoretical mass energy density and scarce lithium resources cannot meet the aforementioned requirements.^[^ ^5^, ^6^ ^]^ At present, the electrode materials of rechargeable secondary batteries are mainly inorganic materials, including layered oxide materials,^[^ ^7^ ^]^ spinel oxides,^[^ ^8^ ^]^ polyphosphates,^[^ ^9^ ^]^ and Prussian blue compounds,^[^ ^10^ ^]^ which usually exhibit high work voltage and rapid redox reaction kinetics. However, the surging battery production has made the stock and cost challenges more prominent for global mineral resources, and the issues are closely associated with the recycled process and non‐hazardous disposal of the discarded batteries as well.^[^ ^11^ ^]^ Alternative sustainable batteries with high mass energy density (such as lithium sulfur (Li‐S) batteries, metal air batteries, organic metal batteries, et al.) are designed to meet higher requirements on the state‐of‐the‐art drones or other energy storage devices.^[^ ^12^, ^13^, ^14^, ^15^ ^]^ A novel strategy is applying other degradable electrode materials, and the organic electrode materials (OEMs) with eco‐friendly and sustainability merits are one type of proper candidates.^[^ ^16^ ^]^ As shown in Figure 1, OEMs have attracted more and more attention owing to their features of environmental friendliness, abundant resource, structural designability, and so on, which make OEMs have broad application prospects in the high‐performance rechargeable batteries. Not only that, some OEMs with flexible structures are not sensitive to the radius and charge of the counter ions, indicating their potentially versatile characteristics for various metal‐ion batteries.^[^ ^17^ ^]^

**FIGURE 2:**
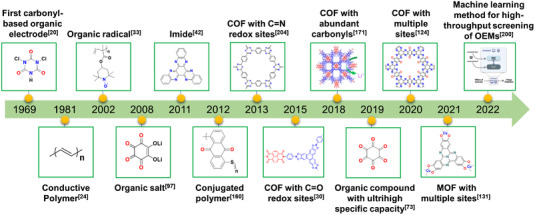
The significant works about OEMs for rechargeable secondary batteries during the past few decades. Reproduced with permission.^[^ ^171^ ^]^ Copyright 2018, WILEY‐VCH GmbH; Reproduced with permission.^[^ ^200^ ^]^ Copyright 2022, Elsevier Inc.

**FIGURE 3:**
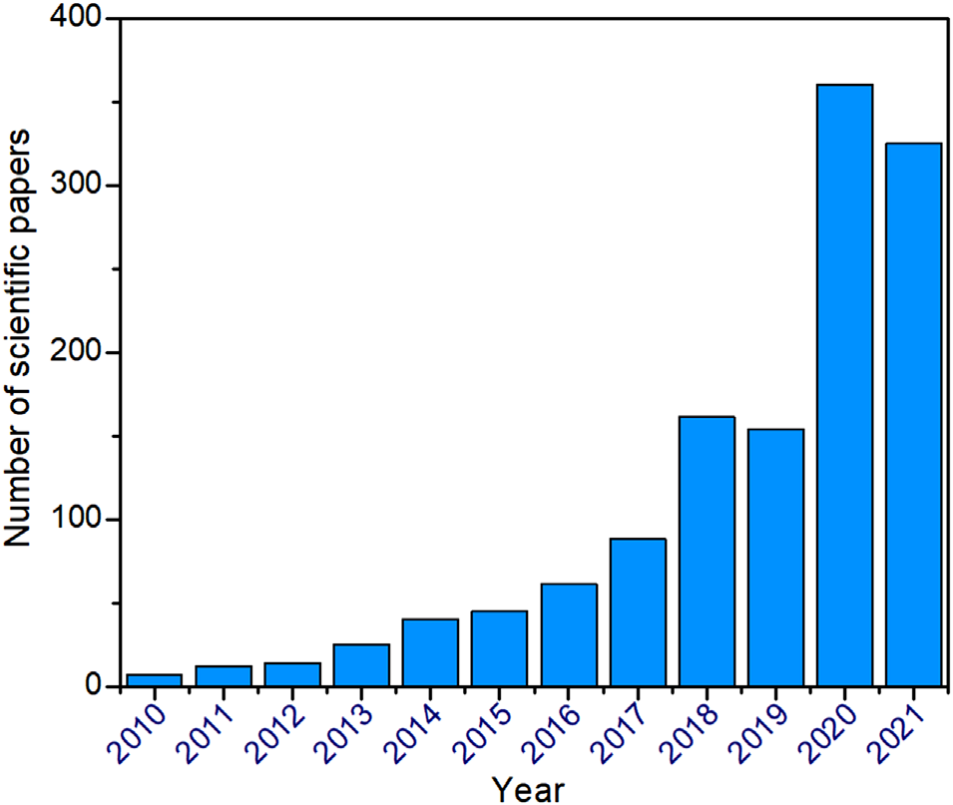
Bar chart of the scientific papers related to OEMs between 2010 and 2021. Data were collected from the ISI Web of Science

Generally, OEMs are connected by covalent bonds and composed of light elements (e.g., C, H, O, N, B, S), and most OEMs could be synthesized easily from accessible raw materials via common organic synthesis reactions.^[^ ^18^, ^19^ ^]^ OEMs emerge as one type of promising candidates for rechargeable batteries early in 1969 (Figure 2), similar to the advent of LIBs.^[^ ^20^ ^]^ Sandstede et al. firstly investigated quinone electrodes with reversible redox reaction property in LIBs, suggesting successful fabrication of the rechargeable secondary batteries.^[^ ^21^ ^]^ Small quinone molecules are mainly troubled by the issue of high solubility in aprotic electrolytes, which could be addressed by porous matrix loading and electrolyte modification methods. As the discovery of conductive polymers in the 1970s, researchers attempted to apply conductive polymers with poor solubility in rechargeable batteries (Figure 2).^[^ ^22^, ^23^, ^24^ ^]^ Conductive polymers (such as polyaniline) show good cycling stability but low capacity, which could be ascribed to their low doping level.^[^ ^25^, ^26^ ^]^ Nevertheless, inspired by the early studying of conductive polymers, the concept of polymerization has been proposed as an effective method to address the solubility issue of small molecules.^[^ ^27^ ^]^ There is a timeline showing the significant works about OEMs during the past few decades (Figure 2), and conjugated polymers with multiple redox‐active groups have been constructed constantly with the rapid development of synthesis technologies.^[^ ^28^, ^29^, ^30^ ^]^ Redox‐active polymers with various functional groups are becoming promising candidates for high‐performance rechargeable batteries, and achieving increasing attention in recent 10 years (Figure 3).

With the booming development of synthesis methods and characterization technologies, OEMs are widely studied in the energy storage systems.^[^ ^31^ ^]^ The redox reaction mechanisms of OEMs are closely related to the types of their redox‐active groups, such as the functional groups of carbonyl, imide, nitroxyl radical, azo, pyrazine, and conductive polymer skeleton.^[^ ^32^, ^33^ ^]^ The stability and reactivity of the organic radical intermediates during the charging/discharging process are crucial to the electrochemical performance of organic batteries, owing to the undesired side reactions of highly reactive intermediates.^[^ ^34^ ^]^ The existing states and structure changes of organic radical intermediates during battery operation need to be further explored at multiple length scales through the real‐time monitor characterization technologies. Notably, operando characterization technologies with high resolution become more and more important for elucidating the capacity fading mechanism of batteries at the molecule level, so as to propose the advanced design strategies for high‐performance pouch‐type cells.^[^ ^35^, ^36^ ^]^ Furthermore, the accurate data interpretation for redox chemistry mechanism and ion transfer path by the theoretical calculations and machine learning methods are crucial for the further exploration of novel OEMs at the microscale level. The deep analysis of redox reaction mechanism aided by the combination of in situ characterization technologies and advanced theoretical calculations is urgent as well.^[^ ^37^ ^]^ With the rapid development of the OEMs, researches would be paid more attention to the inherent issues, high‐throughput screening, effective multiscale mechanism characterizations, and advanced machine learning‐based strategies of future energy storage systems. Moreover, there are a few reviews focusing on the development of the OEMs‐based full batteries,^[^ ^17^ ^]^ which is of significant importance for further exploring eco‐friendly and high‐energy‐density sustainable secondary batteries under various practical working conditions.

Herein, this review is focused on the issues and complex redox mechanisms faced by OEMs from the microscale to macroscale levels, in combination with the state‐of‐art strategies and in‐depth outlook. First, the main parameters for OEMs are provided mainly from five aspects: redox reaction types, redox reaction mechanism (related to the organic radical intermediates), mass energy density, redox reaction potential, and theoretical specific capacity. Next, we present the urgent challenges (including redox potential, theoretical capacity, solubility, electronic conductivity, and complex reaction mechanisms) and advanced strategies of OEMs in organic‐based rechargeable batteries point by point in this review, understanding of high effective methods to improve electrochemical performance of rechargeable organic batteries. Notably, we present a comprehensive introduction to the state‐of‐art characterization technologies, theoretical calculations, and machine learning‐based methods for operando electrochemical reactions in various metal‐ion batteries in this review. Finally, we have proposed the design approaches of OEMs‐based full cells and outlook of OEMs for future rechargeable organic batteries as well, so as to promote the commercial application of sustainable organic batteries.

## 2 REDOX REACTION CHEMISTRY

### 2.1 Redox reaction types of OEMs

**FIGURE 4:**
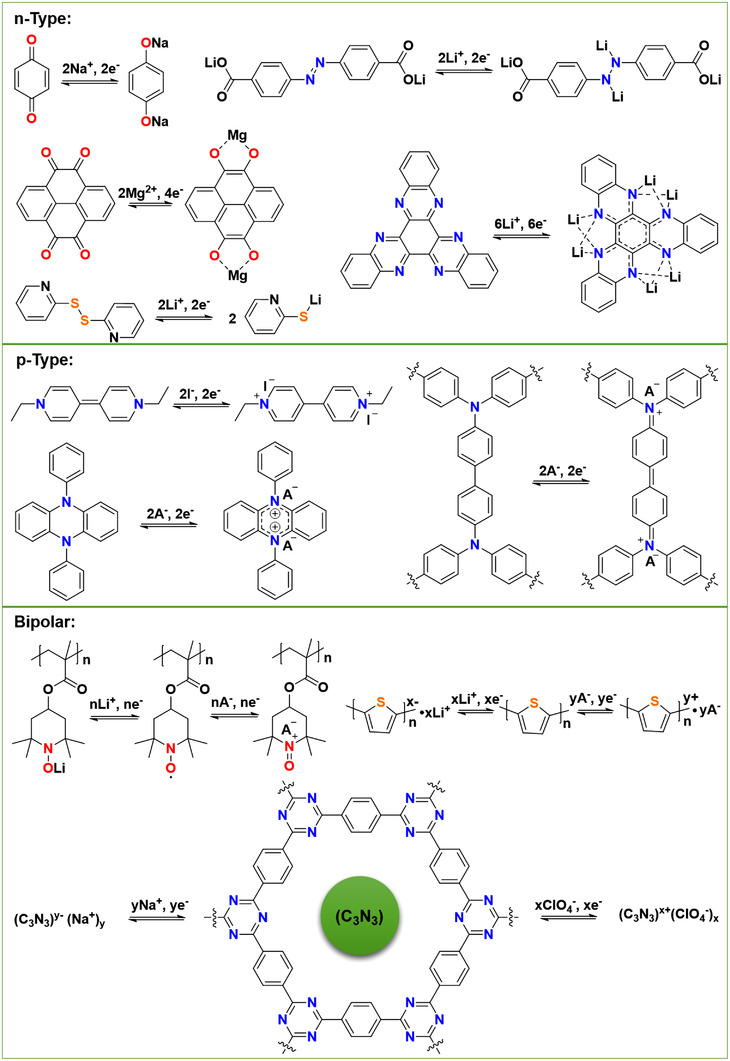
Categories of common OEMs and the corresponding redox reaction mechanisms

Generally, the redox reaction mechanism of OEMs is closely related to the charge‐state changes of their electroactive groups, which corresponds to the types of the active functional groups (C═O, C═N, C═C, N═N, C≡C, S─S, nitroxyl radicals, conductive polymer skeleton, etc.) at the molecule level.^[^ ^38^ ^]^ The redox reaction types could be classified into n‐type, p‐type, and bipolar type ion storage processes (Figure 4), corresponding to a cation incorporation, an anion uptake process, and either cation or anion combination, respectively.^[^ ^39^ ^]^

**FIGURE 5:**
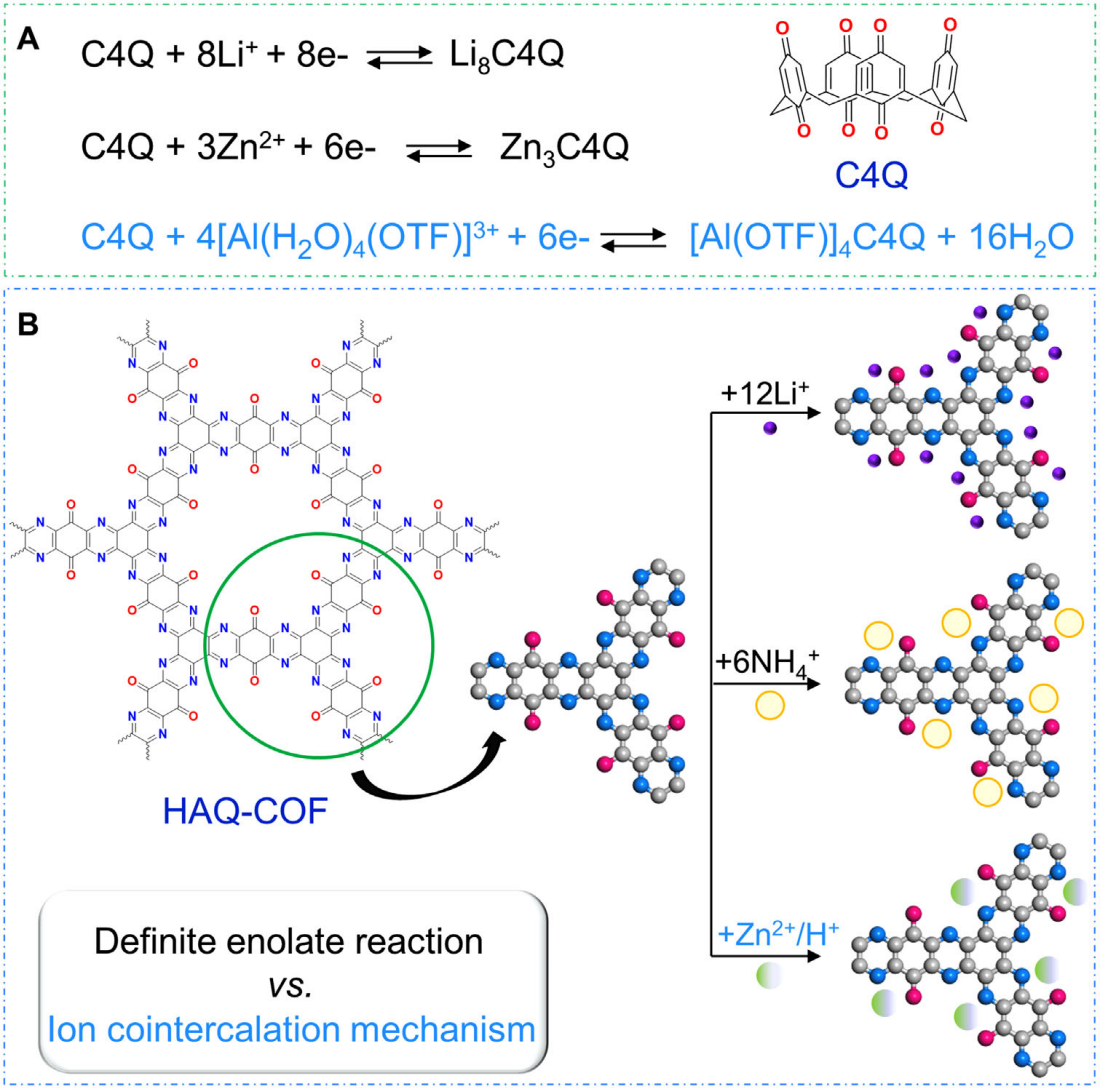
(A) The ion intercalation/extraction process of C4Q molecule in different rechargeable batteries. (B) The illustration of different ion storage mechanisms for per HAQ‐COF repeating unit

Quinones, imides, azos, and amides are classical kinds of n‐type OEMs, which are usually undergoing reversible enolization between active sites and cations (e.g., Li^+^, H^+^, NH_4_^+^, Na^+^, K^+^, Zn^2+^, Mg^2+^, Al^3+^).^[^ ^40^, ^41^ ^]^ Generally, many n‐type OEMs with multi‐electron redox mechanism show high specific capacity, drawing increasing attention as a promising alternative to conventional inorganic electrodes.^[^ ^42^ ^]^ Unlike the definite enolate reactions between alkali metal ions and redox‐active sites, multivalent ions may experience anion cointercalation chemistry during the ion intercalation/extraction process, owing to the high charge densities of multivalent ions. For instance, one calix[4]quinone (C4Q) molecule can combine with eight Li^+^ ions, indicating that per active carbonyl site is bound to one alkali metal ion.^[^ ^43^ ^]^ However, as shown in Figure 5A, C4Q can combine with no more than four multivalent ions (Mg^2+^ or Zn^2+^) owing to the charge density and steric hindrance factors, and even less for that of [Al(OTF)]^2+^, which is ascribed to the strong coulombic interaction of Al^3+^ with anions in the electrolyte.^[^ ^44^, ^45^ ^]^

The p‐type OEMs (such as phenazine (PZ) and carbazole derivatives) can interact with anions (such as PF_6_^–^, ClO_4_^–^, I^–^, BF_4_^–^, TFSI^–^) after their oxidized reaction, and they usually exhibit higher discharge potential.^[^ ^46^ ^]^ The recently reported phenothiazine‐based cathode material displays an average redox voltage of 3.7 V (vs. Li^+^/Li), which is comparable to the conventional inorganic cathode materials.^[^ ^47^ ^]^ In contrast to the n‐type OEMs, the redox reactions of p‐type OEMs involve the compensation process of counter anions, potentially showing faster kinetics of redox reactions.^[^ ^48^ ^]^ However, most p‐type electrodes with limited anionic doping degree show low discharge capacity and complex redox mechanism, which hinders the further research of p‐type OEMs in portable metal‐ion batteries.^[^ ^49^, ^50^ ^]^ Notably, p‐type OEMs with anion redox chemistry draw increasing attention as a promising electrode in stationary‐type batteries, owing to the merits of high redox voltage and fast rate capability.

In contrast, bipolar type OEMs (e.g., nitroxyl radicals, polyaniline, polypyrrole, and conductive polythiophene skeleton) could be reduced or oxidized at different output voltage ranges, and they have both n‐type and p‐type redox features for per redox‐active molecule.^[^ ^51^, ^52^ ^]^ For example, Chen's research group integrated maximum n‐type and p‐type redox‐active moieties into one stable polymer to achieve high‐performance bipolar type OEMs with different features, namely high capacity or high redox voltage.^[^ ^53^ ^]^ In terms of the assembly of all‐organic full cells, bipolar organic materials are usually regarded as the cathode materials in rechargeable batteries due to their high p‐doping discharge potentials, and the redox‐active n‐type moieties are used as the anode. Recently, growing attention is paid to the bipolar type OEMs with high energy density and eco‐friendly features.

### 2.2 Redox reaction mechanism

Considerable attention has been paid to design novel OEMs, while the in‐depth exploration of the redox reaction mechanism of organic compound is of significant guidance for the rapid development of organic rechargeable batteries.^[^ ^54^, ^55^ ^]^ The ion uptake/release processes of OEMs are still elusive during the battery operation at multiple scales, and the stability and reactivity of the organic intermediates during the charging/discharging process are crucial to the electrochemical performance of rechargeable organic batteries. Similar to the above ion intercalation/extraction processes for C4Q molecule, the redox mechanism of a two‐dimensional covalent organic framework (COF) material with multiple redox‐active groups (C═O and C═N), namely as HAQ‐COF, also suggests the evident difference of redox mechanisms for different ion storage processes. As shown in Figure 5B, it can storage 12 Li^+^ ions upon one repetitive unit,^[^ ^56^ ^]^ while the repetitive HAQ‐COF unit combines with 6 NH_4_^+^ ions via the hydrogen bond chemistry, owing to the solvation behavior of NH_4_^+^.^[^ ^57^ ^]^ Besides, this two‐dimensional HAQ‐COF experiences a co‐intercalation process of Zn^2+^ and H^+^ in the aqueous zinc‐ion batteries,^[^ ^58^ ^]^ demonstrating the enhanced Zn^2+^ storage of carbonyl groups in the HAQ‐COF electrodes. The in‐depth understanding regarding redox reactions of OEMs by high resolution technologies or advanced computational simulations is still a challenge for the construction of high‐performance organic batteries.^[^ ^59^ ^]^

### 2.3 Mass energy density

Mass energy density is crucial for the development of a driving range of ∼500 km for current electric vehicles, while the mass energy density of the present commercial LIBs is far below the future energy density target (500 Wh kg^–1^).^[^ ^60^ ^]^ The theoretical mass energy density of the entire batteries is determined by the theoretical specific capacity and redox reaction potential as shown in the following equation:

(1) w=Q×E

where *w* is the theoretical mass energy density, *Q* and *E* are the theoretical specific capacity and redox reaction potential of the entire batteries, respectively. The basic requirements for achieving the targeting mass energy density mainly include high theoretical capacity and redox reaction potential of the rechargeable secondary batteries.^[^ ^61^, ^62^ ^]^

### 2.4 Theoretical specific capacity

**FIGURE 6:**
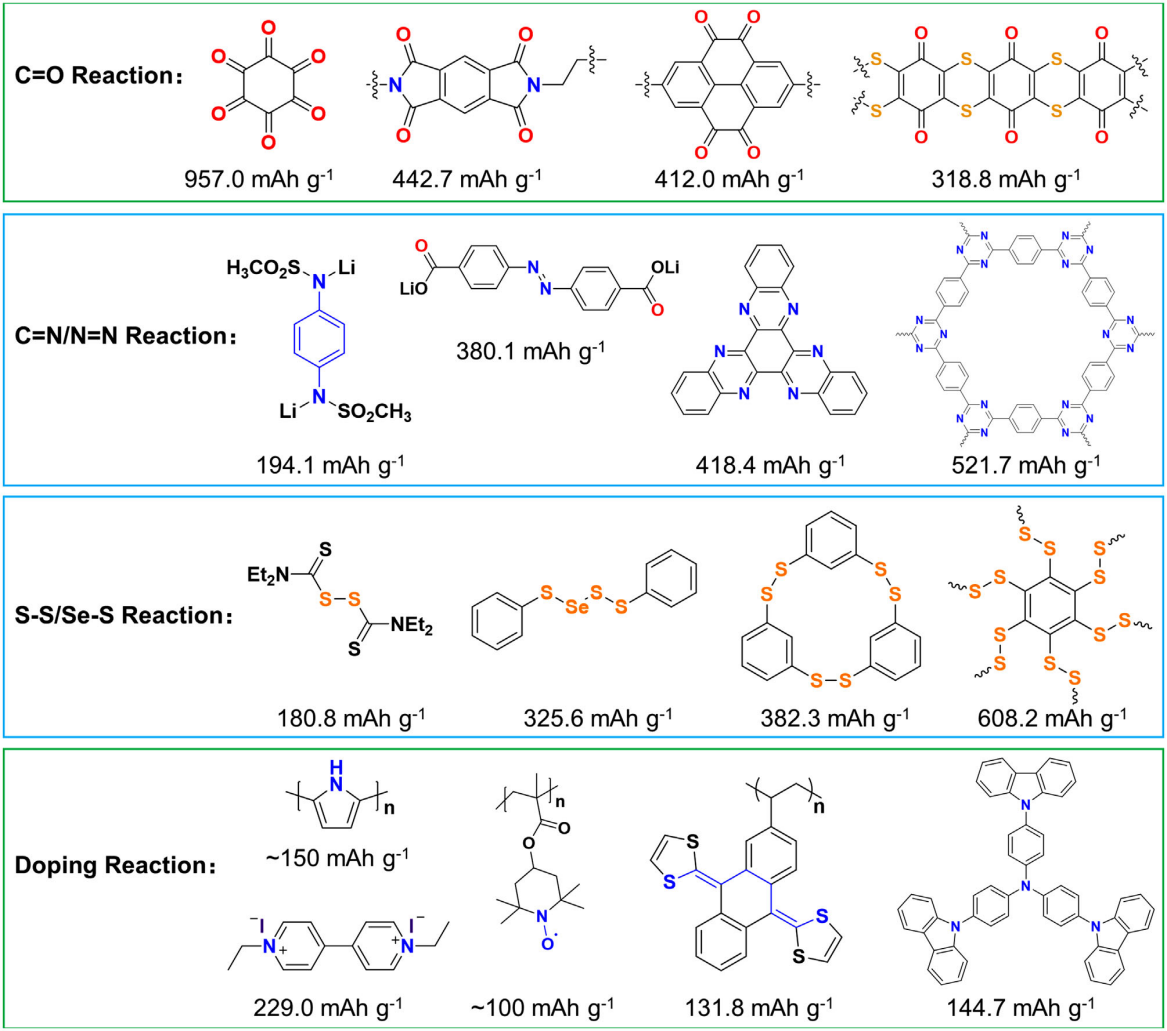
Common OEMs with different reaction types

OEMs are usually designed with the merits of high capacity, highly reversible reaction, and low cost. Common OEMs with different reaction types and theoretical specific capacities are shown in Figure 6. The theoretical capacity for an OEM could be calculated as follows.

(2) $$Q=nF/3.6M_{w}$$

where *M*_w,_ *n*, and *F* are molecular weight, transferred electrons number per molecule, and Faraday constant, respectively. Based on the above formula, increasing the electron transfer number or the content of active ingredients of OEMs can be used to improve their theoretical mass energy density. Some important electrochemical parameters of different types of organic electrodes are listed in Table 1, and most of them show high actual discharge capacity, owing to the structural engineering strategy for the OEMs.

**TABLE 1:** Important electrochemical parameters (i.e., operating voltage window, discharge capacity, cycling stability, rate capability) of typical organic electrodes

Organic electrodes	Chemical structure	Active loading	Operating voltage window	Discharge capacity	Cycling stability	Rate capability
Me_2_BBQ^[^ ^100^ ^]^	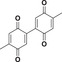	30 wt%	1.8–3.4 V vs. Li^+^/Li	0.005 A g^–1^, 332 mAh g^–1^	0.3 A g^–1^, 2,000th, 82.9%	0.6 A g^–1^, 122 mAh g^–1^
Na_2_C_6_O_6_^[^ ^102^ ^]^	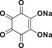	80 wt%	0.5–3.3 V vs. Na^+^/Na	0.05 A g^–1^, 484 mAh g^–1^	0.5 A g^–1^, 50th, 90.8%	1.0 A g^–1^, 371 mAh g^–1^
C_6_O_6_^[^ ^73^ ^]^	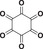	50 wt%	1.2–4.0 V vs. Li^+^/Li	0.02 A g^–1^, 902 mAh g^–1^	0.05 A g^–1^, 100th, 80%	0.5 A g^–1^, 382 mAh g^–1^
HATN^[^ ^75^ ^]^	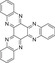	30 wt%	1.2–3.9 V vs. Li^+^/Li	0.4 A g^–1^, 395 mAh g^–1^	8.0 A g^–1^, 10,000th, ∼70%	8.0 A g^–1^, 222 mAh g^–1^
Di‐TEMPO^[^ ^166^ ^]^	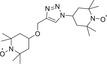	50 wt%	−0.05–0.8 V vs. Fc^+^/Fc	0.066 A g^–1^, 117 mAh g^–1^	0.13 A g^–1^, 200th, 68.4%	0.66 A g^–1^, 50 mAh g^–1^
PAN^[^ ^25^ ^]^	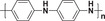	70 wt%	2.0–4.0 V vs. K^+^/K	0.01 A g^–1^, 138 mAh g^–1^	0.05 A g^–1^, 100th, 98%	0.2 A g^–1^, 95 mAh g^–1^
PBQS^[^ ^116^ ^]^	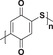	60 wt%	1.5–4.0 V vs. Li^+^/Li	0.05 A g^–1^, 275 mAh g^–1^	0.5 A g^–1^, 1,000th, 86%	5.0 A g^–1^, ∼200 mAh g^–1^
P14AQ^[^ ^119^ ^]^	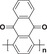	60 wt%	1.5–3.0 V vs. Li^+^/Li	0.526 A g^–1^, 263 mAh g^–1^	0.526 A g^–1^, 1000th, 99.4%	5.26 A g^–1^,181.5 mAh g^–1^
PTO^[^ ^88^ ^]^	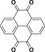	60 wt%	0.4–1.5 V vs. Zn^2+^/Zn	0.04 A g^–1^, 336 mAh g^–1^	3.0 A g^–1^, 1,000th, 70%	20 A g^–1^, 113 mAh g^–1^
BPOE^[^ ^204^ ^]^	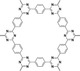	70 wt%	1.3–4.1 V vs. Na^+^/Na	0.01 A g^–1^, ∼200 mAh g^–1^	1.0 A g^–1^, 7,000th, 80%	0.1 A g^–1^, ∼120 mAh g^–1^
PHATN^[^ ^125^ ^]^	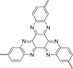	60 wt%	0.5–2.3 V vs. Mg^2+^/Mg	0.02 A g^–1^, 146 mAh g^–1^	0.02 A g^–1^, 200th, 75.3%	0.146 A g^–1^, 60 mAh g^–1^
4KT‐Tp COF^[^ ^126^ ^]^	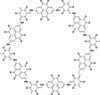	37 wt%	0–0.6 V vs. Ag^+^/Ag	0.2 A g^–1^, 583 F g^–1^	5.0 A g^–1^, 20,000th, 92%	10 A g^–1^, 152 F g^–1^
Co‐HAB^[^ ^155^ ^]^	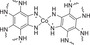	90 wt%	0.5–3.0 V vs. Na^+^/Na	0.05 A g^–1^, 291 mAh g^–1^	4.0 A g^–1^, 150th, ∼100%	12 A g^–1^, 152 mAh g^–1^

### 2.5 Redox reaction potential

**FIGURE 7:**
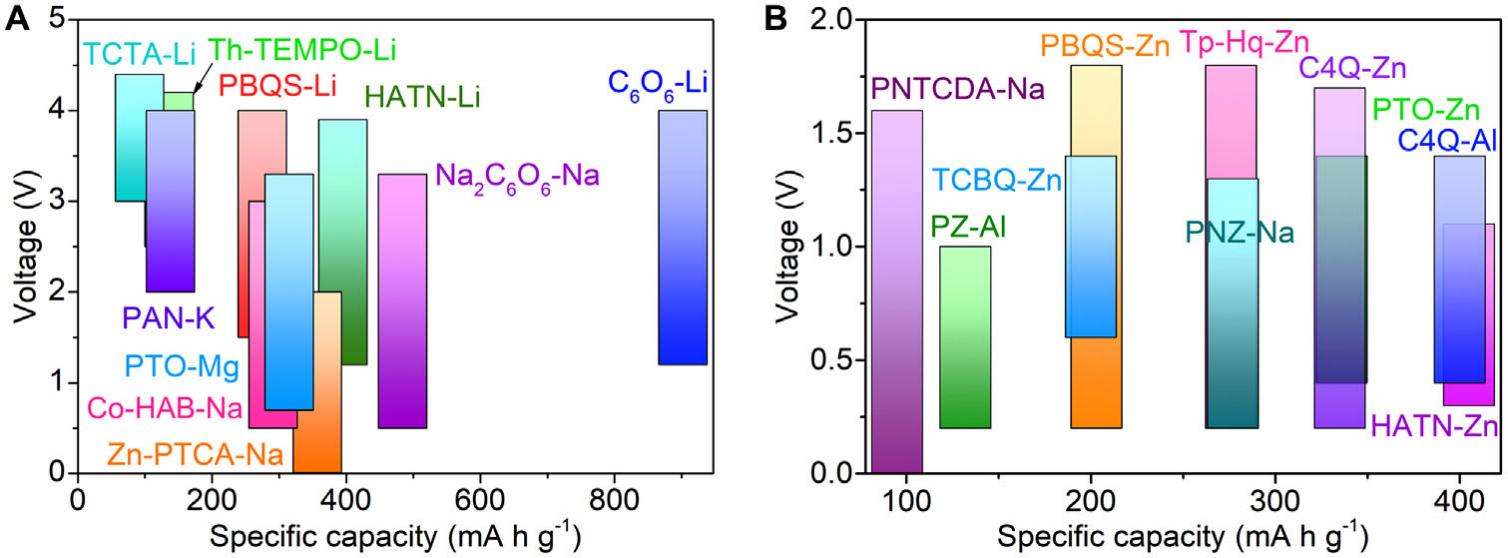
Comparison of working voltage window and discharge capacity of different types of OEMs in the organic electrolyte (A) and the aqueous electrolyte (B). The structures or names of most organics can be found in Table 1 and the following sections

The redox reaction potential, mainly related to the types of the active functional groups, substituent groups, and conjugated structure, is another factor that affects the mass energy density of OEMs.^[^ ^63^ ^]^ According to the density functional theory (DFT) calculations, the reduction potential of OEMs is closely correlated with the energy level of the lowest unoccupied molecular orbital (LUMO) of organic molecules, as well as the electron affinity.^[^ ^64^ ^]^ Thus, the factors affecting the electron affinity or the LUMO energy level of redox‐active sites of OEMs could be taken into consideration to adjust the redox potential at the molecule level. Notably, with the merits of high‐throughput screening and function‐oriented programming, machine learning‐assisted methods are applied to predict novel OEMs with ideal potentials.^[^ ^65^ ^]^ The redox potential of OEMs could be tuned mainly from the following four aspects: (1) the type of redox‐active sites; (2) conjugated structure; (3) the type of electron‐withdrawing/donating groups (such as ─F or ─NH_2_); (4) other structure changes. With rational design of organic molecule structure, the theoretical mass energy density of some OEMs has been improved to >1000 Wh kg^–1^ at the coin‐type battery level, far beyond that of the conventional in OEMs.^[^ ^44^ ^]^ The comparation of working voltage window and discharge capacity of different types of OEMs is shown in Figure 7. The working voltage windows of OEMs are around 3.0 V in the organic electrode, and <2.0 V in the aqueous electrolyte. The combination of redox potential tunability and function‐oriented structural engineering is indispensable for the development of high‐energy‐density rechargeable batteries.

## 3 CHALLENGES AND STRATEGIES FOR OEMS

### 3.1 Challenges for OEMs

OEMs mainly suffer from the issues of high solubility in aprotic electrolytes, poor electronic conductivity, lower redox potential, and so on, leading to capacity fading and low energy density for rechargeable batteries.^[^ ^26^ ^]^ Chemical reversibility and reaction kinetics of OEMs have an important effect on the electrochemical performance of the overall batteries.^[^ ^66^ ^]^ Challenges in data collection and interpretation for rechargeable batteries are closely related to the composite change, structure transformation, and chemistry mechanism, especially for defects chemistry of crystalline conjugated compounds or organic salts.^[^ ^67^, ^68^ ^]^ The challenges for OEMs are shown as follows:

Theoretical capacity and redox potential: The mass energy densities of OEMs are closely related to their theoretical capacity and redox potential. On the one hand, most of organic molecules with much inactive content always show low theoretical specific capacity, and the poor conductivity and the agglomeration behavior further reduce their actual capacity. On the other hand, the working potential of OEMs is affected by the active site type, conjugated structure, substituent groups, and other structural changes. How to increase the output capacity and working voltage of OEMs are challenges for the construction of high‐energy‐density rechargeable batteries.

Solubility: Generally, small molecules and their discharge products are always faced with solubility problem in the common organic or aqueous electrolytes. The solubility issue is mainly attributed to the similar polarity between the small molecules and the electrolytes, resulting in the deteriorating capacity and poor cycling performance for OEMs‐based rechargeable batteries.

Electronic conductivity: Most OEMs even with good structural stability still suffer from poor intrinsic electronic conductivity, reducing the utilization of active sites and rate capability of the overall batteries.^[^ ^69^ ^]^ The ion/electron transfer efficiency has a great effect on the electrochemical performance of OEMs as well. Adding conductive carbon is a direct way to enhance the electron conductivity of organic electrode. How to improve the intrinsic electronic conductivity of OEMs through structural engineering strategy is still a big challenge.

Complex redox mechanism: The ion uptake/release processes of OEMs are still elusive during the battery operation, and most of them are related to the generation of organic radical intermediates.^[^ ^70^, ^71^ ^]^ The stability and reactivity of the organic intermediates during the charging/discharging process are crucial to the stability of organic batteries, owing to the undesired side reactions of the highly reactive intermediates.^[^ ^72^ ^]^ The structure characterization of organic intermediates by high resolution technologies or advanced computational simulations is still a challenge for the construction of high‐performance organic batteries.

### 3.2 Strategies for the given issues of OEMs

**FIGURE 8:**
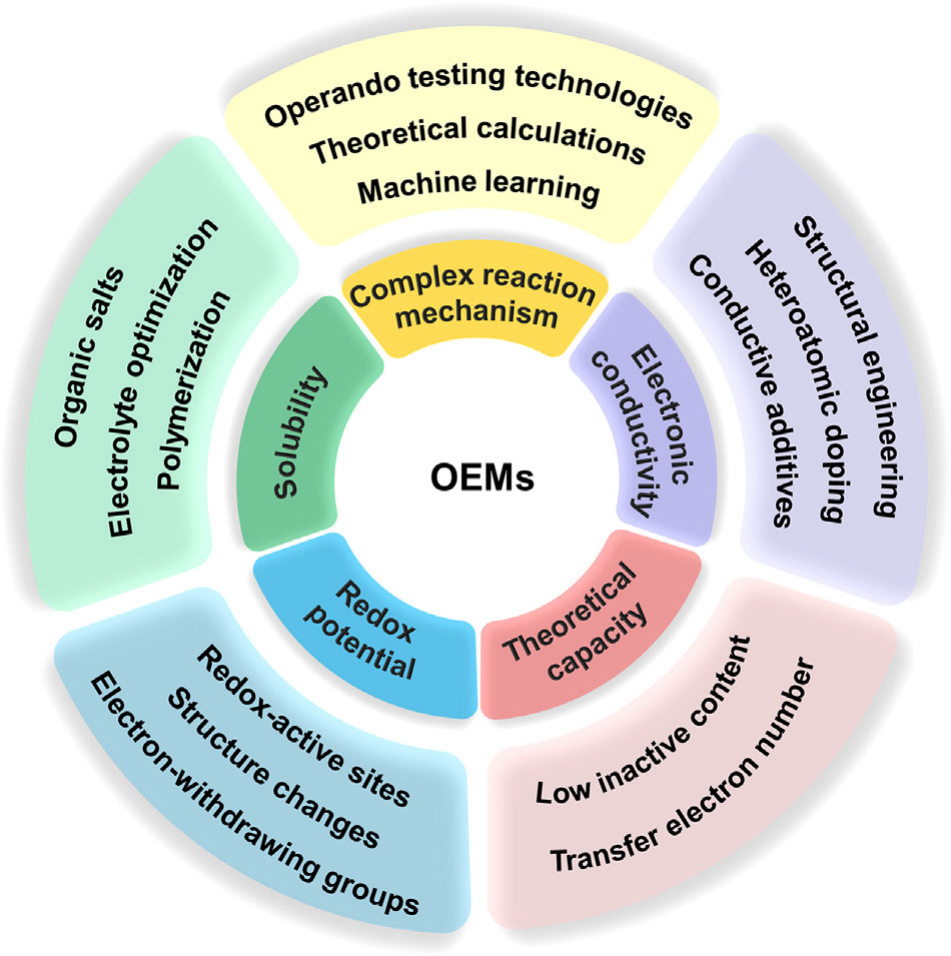
Challenges (including redox potential, theoretical capacity, solubility, electronic conductivity, and complex redox mechanism) and the corresponding strategies of the OEMs

The corresponding strategies for current issues faced by OEMs are carefully discussed point by point, including redox potential, theoretical capacity, solubility, electronic conductivity, and complex redox mechanism. The redox potential and theoretical capacity of OEMs could be tuned by the structural engineering strategy, and the solubility and electronic conductivity issues are potentially addressed by the function‐oriented design for organic molecule structures and electrode materials. In addition, the exploration for the complex redox mechanism of the organic‐based batteries requires the support of operando testing technologies, advanced theoretical calculations, and even the machine learning‐based methods. This review mainly presents the challenges faced by OEMs and the possible solutions to per issue from the following five aspects (Figure 8).

#### 3.2.1 Strategies to improve the theoretical capacity of OEMs

**FIGURE 9:**
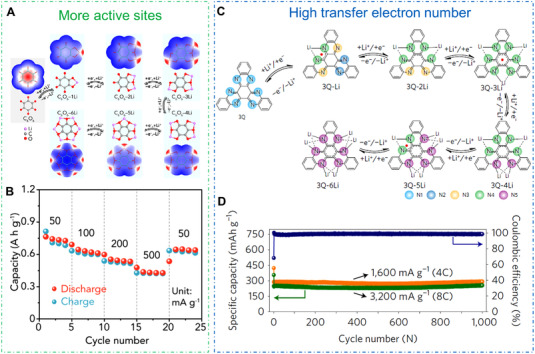
(A) Lithium storage mechanism and (B) rate capability of C_6_O_6_ electrode within an ionic liquid electrolyte. Reproduced with permission.^[^ ^73^ ^]^ Copyright 2019, WILEY‐VCH GmbH. (C) Lithium ion uptake/release mechanism and (D) cycling performance of 3Q electrode. Reproduced with permission.^[^ ^75^ ^]^ Copyright 2017, Nature Publishing Group

According to the above Equation (2) in Section 2, the theoretical capacity of OEMs is mainly related to their electron transfer number and the proportion of active ingredients. To achieve higher actual capacity of OEMs, on the one hand, structural engineering should take into consideration to reduce the inactive ingredients in organic molecule, improving the intrinsic theoretical capacity of OEMs. On the other hand, the structure modification of organic electrode is indispensable for full utilization of active sites. Chen et al. synthesized a cyclohexanehexone compound (C_6_O_6_) through the dehydration reaction in Figure 9A.^[^ ^73^ ^]^ The C_6_O_6_ molecule contains six symmetrical carbonyls, exhibiting an ultrahigh theoretical capacity of 957.0 mAh g^–1^ (Figure 9B). Although the average discharge voltage of the LIBs was about 1.7 V (vs. Li^+^/Li), the C_6_O_6_ cathode displayed a high mass energy density of 1,533.0 Wh kg^–1^, which was much higher than those of the reported organic cathode materials. Zhang et al. reported a boroxine‐linked PTO‐COF as binder‐free cathode material, and per PTO‐COF unit showed abundant redox‐active carbonyls on the conjugated structure, which made the PTO‐COF cathode show superb structural/chemical stability and an energy density of 534.6 Wh kg^–1^ (198 mAh g^–1^ × 2.7 V (vs. Li^+^/Li)).^[^ ^74^ ^]^ Loh et al. constructed a fused N‐heteroaromatic triquinoxalinylene (3Q) electrode in rechargeable LIBs in Figure 9C.^[^ ^75^ ^]^ The 3Q molecule featured six of electron‐deficient pyrazine sites on the hexaazatrinaphthalene‐based π‐conjugated structure, and the 3Q electrode displayed an output capacity of 395 mAh g^–1^ and excellent cycling stability (Figure 9D). The high‐electron‐deficient organic molecules with multiple redox sites are promising electrodes for the high‐capacity organic rechargeable batteries.

#### 3.2.2 Strategies to enhance the redox potential of organic cathode materials

Generally, the redox reaction potentials of the organic cathode materials are around 1.5–4 V vs. Li^+^/Li, lower than that of the traditional inorganic cathodes.^[^ ^76^, ^77^ ^]^ The redox reaction potential of the OEMs could be slightly regulated by introducing electron‐withdrawing groups (─F, ─Cl, ─Br, ─COOH, ─CN, ─NO_2_) or electron‐donating groups (─OH, ─NH_2_, ─OCH_3_) into their conjugated structures.^[^ ^78^ ^]^ Frayret et al. tuned the redox voltages of quinonic species by altering their functional groups, confirming the delocalized‐localized effect of derivatives on tuning their redox potentials (even enabling 2.0 V vs. Li^+^/Li) by the DFT calculations.^[^ ^79^ ^]^ Nevertheless, the introduction of the inactive substituents leads to limited effect on redox voltage tunability of OEMs.^[^ ^80^ ^]^

**FIGURE 10:**
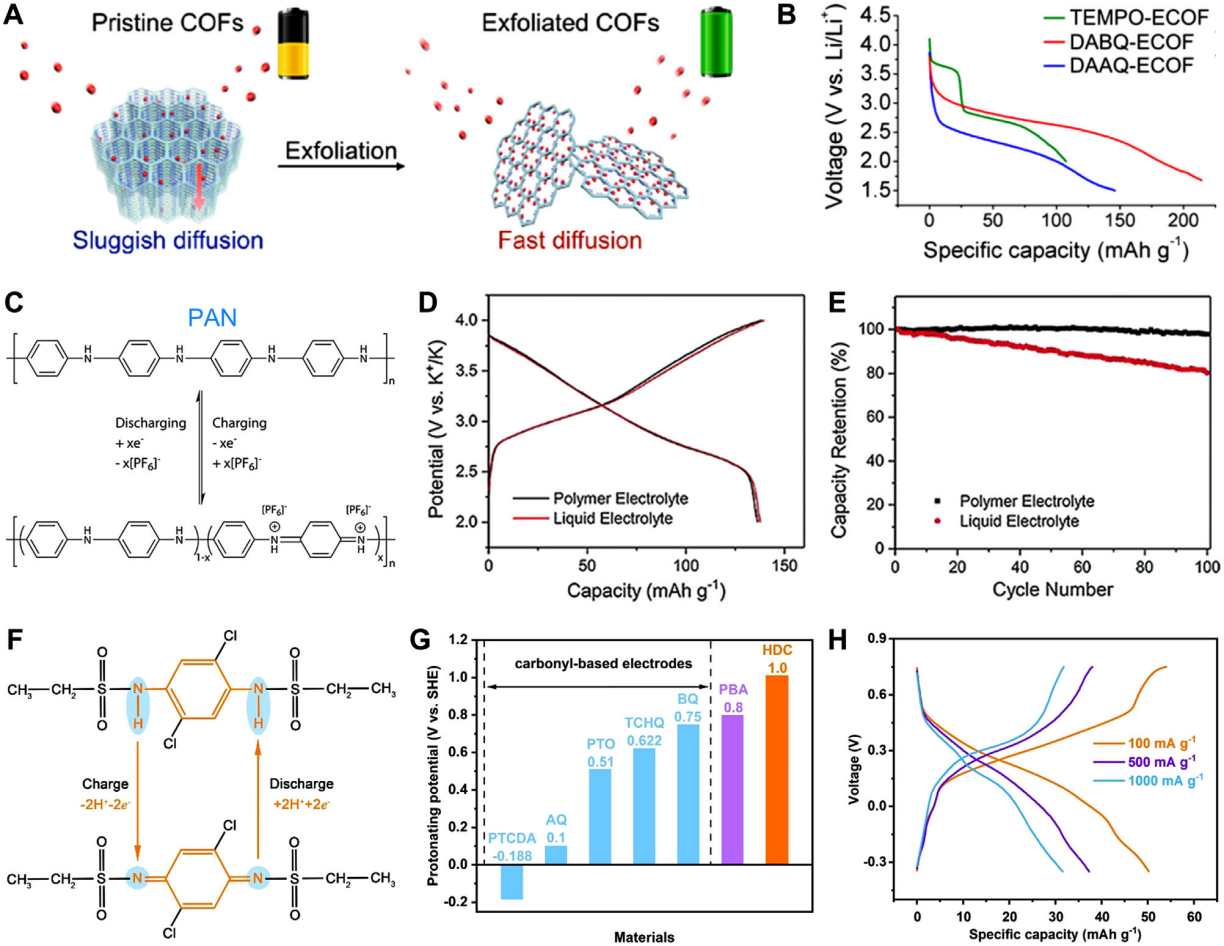
(A) The schematic illustration of exfoliated COFs‐based cathode for LIBs. (B) The discharge curves of these three distinct redox‐active COFs electrodes at 0.02 A g^–1^. Reproduced with permission.^[^ ^81^ ^]^ Copyright 2017, The American Chemical Society. (C) The redox reaction mechanism of the polyaniline cathode with a polymer‐gel electrolyte. The discharge/charge curves (D) and rate performance (E) of the polyaniline cathode with liquid and polymer electrolytes. Reproduced with permission.^[^ ^25^ ^]^ Copyright 2018, WILEY‐VCH GmbH. (F) The redox mechanism of HDC electrode. (G) Working potential of HDC and other reported OEMs. (H) Charge and discharge curves of the full battery with HDC cathode and activated carbon anode. Reproduced with permission.^[^ ^82^ ^]^ Copyright 2022, WILEY‐VCH GmbH

Additionally, the redox potential is related to the redox reaction types of the redox‐active sites of OEMs, thus choosing OEMs with different redox‐active sites for the proper battery systems is an efficient way to regulate their redox potentials. Wang's research group reported three distinct redox‐active COFs with 2D few‐layer nanosheets (Figure 10A), and by altering the types of the redox‐active sites, they showed different voltage plateaus, such as 2.8 and ∼3.0 V (vs. Li^+^/Li) for the anthraquinone‐based and tempo‐based cathodes in Figure 10B, respectively.^[^ ^81^ ^]^ Goodenough et al. developed a high‐energy‐density potassium‐ion battery with polymer‐gel electrolyte of cross‐linked poly(methyl methacrylate) and a polyaniline cathode in Figure 10C, and the p‐type polyaniline cathode exhibited a working potential of ∼3.0 V vs. K^+^/K and excellent cycling stability (Figure 10D,E).^[^ ^25^ ^]^ Lu et al. presented 2,5‐dichloro‐1,4‐phenylene‐*bis*((ethylsulfonyl) amide) (HDC) with nitrogen active centers and electron‐withdrawing groups (Figure 10F) used as the cathode for quasi‐solid proton‐ion battery, and HDC electrode showed a high output potential of ∼1.0 V (vs. SHE) in Figure 10G.^[^ ^82^ ^]^ The full battery with HDC cathode and activated carbon anode displayed a reversible capacity of ∼50 mAh g^–1^ and good rate capability (Figure 10H), exhibiting the effective regulation of redox potentials of OEMs by the structural engineering strategy.

**FIGURE 11:**
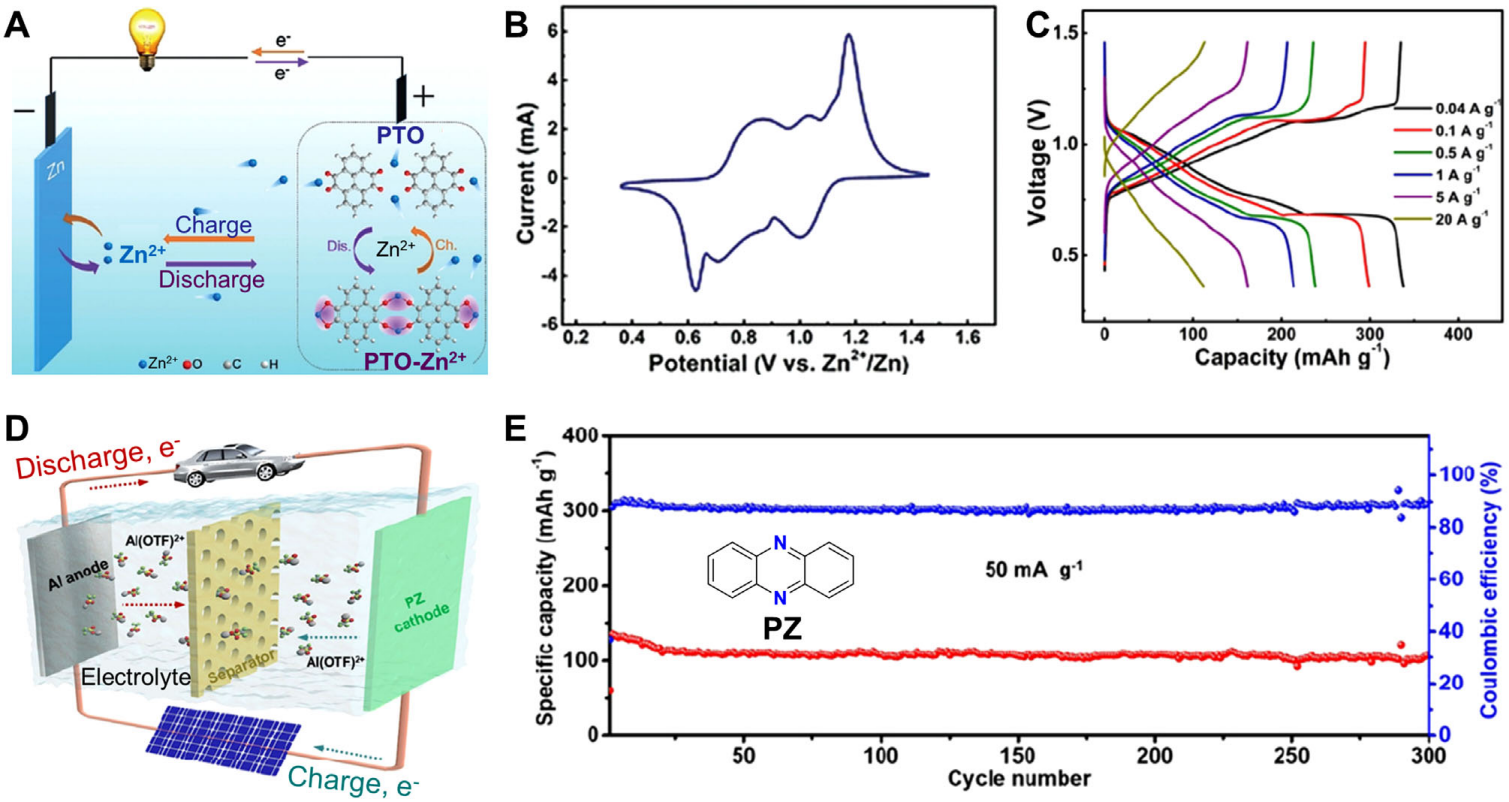
(A) Schematic redox reaction mechanism of aqueous PTO//Zn battery. (B) CV curve at 1.0 mV s^–1^ and (C) galvanostatic discharge/charge profiles at different current densities of PTO electrode. Reproduced with permission.^[^ ^88^ ^]^ Copyright 2018, WILEY‐VCH GmbH. (D) Illustration of reversible reaction mechanism of aqueous aluminum battery using PZ cathode. (E) Cycling performance of PZ cathode at the current density of 0.05 A g^–1^. Reproduced with permission.^[^ ^89^ ^]^ Copyright 2021, WILEY‐VCH GmbH

Moreover, aqueous batteries have the advantages of high safety, environmentally friendliness, and high ionic conductivity, but the narrow electrochemical stability window (1.23 V) and heavy side reactions of aqueous batteries limit their applications in large‐scale energy storage systems.^[^ ^83^, ^84^, ^85^ ^]^ Notably, the moderate redox potentials of organic materials locate in the voltage window of aqueous batteries, providing potential applications for OEMs with high capacity, flexibility, and sustainability.^[^ ^86^, ^87^ ^]^ For example, Wang et al. presented a rechargeable zinc battery with a PTO‐based cathode in Figure 11A, which exhibited a high specific capacity of 336 mAh g^–1^ for reversible Zn^2+^ storage and a high energy density of 186.7 Wh kg^–1^ for PTO//Zn pouch‐type cell (Figure 11B,C).^[^ ^88^ ^]^ Besides, Guo and co‐workers used PZ with redox‐active moieties as cathode material in aqueous aluminum batteries (Figure 11D). The PZ electrode with rigid structure and co‐intercalation behavior showed a reversible capacity of 139 mAh g^–1^ and good cycling stability (Figure 11E).^[^ ^89^ ^]^ The anion co‐intercalation chemistry of PZ material lays the foundation for the development of multivalent‐ion battery systems. Furthermore, Sjödin et al. successfully constructed an all‐organic proton battery via conducting poly(3,4‐ethylenedioxy‐thiophene) functionalized with anthraquinone and benzonquinone groups.^[^ ^90^ ^]^ The proton battery with proper working voltage window delivered excellent reversibility for more than 1,000 cycles, suggesting the feasibility of assembling all‐organic proton batteries without metal ions and conductive additives. Therefore, OEMs are one type of the most promising candidates for aqueous rechargeable batteries in future grid‐scale energy storage devices.

#### 3.2.3 Strategies to inhibit the solubility of small organic molecules

In general, small organic molecules are likely to dissolve in the aprotic (ester‐ or ether‐based) electrolytes. The soluble organic molecules shuttle to metal anode due to the concentration gradient, leading to the corrosion of active metal and the rapid capacity deterioration.^[^ ^91^, ^92^, ^93^ ^]^ It is worth noting that the carbon‐coated adsorption strategy could inhibit the diffusion of small molecules through the physical confinement or physical and chemical adsorption interactions, while this strategy has a limited effect on blocking the shuttling of small molecules. The effective strategies for inhibiting the solubility of organic small molecules are including regulating the structure of OEMs, optimizing the electrolyte, polymerization, and so on.^[^ ^94^ ^]^

First, by means of the structural engineering at the molecular level, using organic salts instead of the soluble small molecules as electrode materials is a common strategy. Some organic salts are poorly soluble in the aprotic electrolyte owing to the large polarity differences.^[^ ^95^, ^96^ ^]^ Tarascon and colleagues first reported dilithium rhodizonate used as the cathode for rechargeable batteries, which showed a high discharge capacity of ∼500 mAh g^–1^ but inferior cycle performance.^[^ ^97^ ^]^ Chen's group used a (Na_4_DHTPA) as both electrodes for the all‐organic full batteries, which exhibited a coulombic efficiency of ∼100% after 100 cycles.^[^ ^98^ ^]^ Xu et al. presented an insoluble *N*,*N*′‐*bis*(glycinyl) naphthalene diimide, this organic salt‐based electrode demonstrated a superb cycling performance (a capacity retention of 57.3% after 70,000 cycles) in ether‐based electrolytes.^[^ ^99^ ^]^ However, recent researches show that some organic salts still experience inferior cycling stability in the aprotic electrolyte due to their structure changes during the battery operation, which suggests the limitation of the organic salts strategy.^[^ ^100^, ^101^, ^102^ ^]^

**FIGURE 12:**
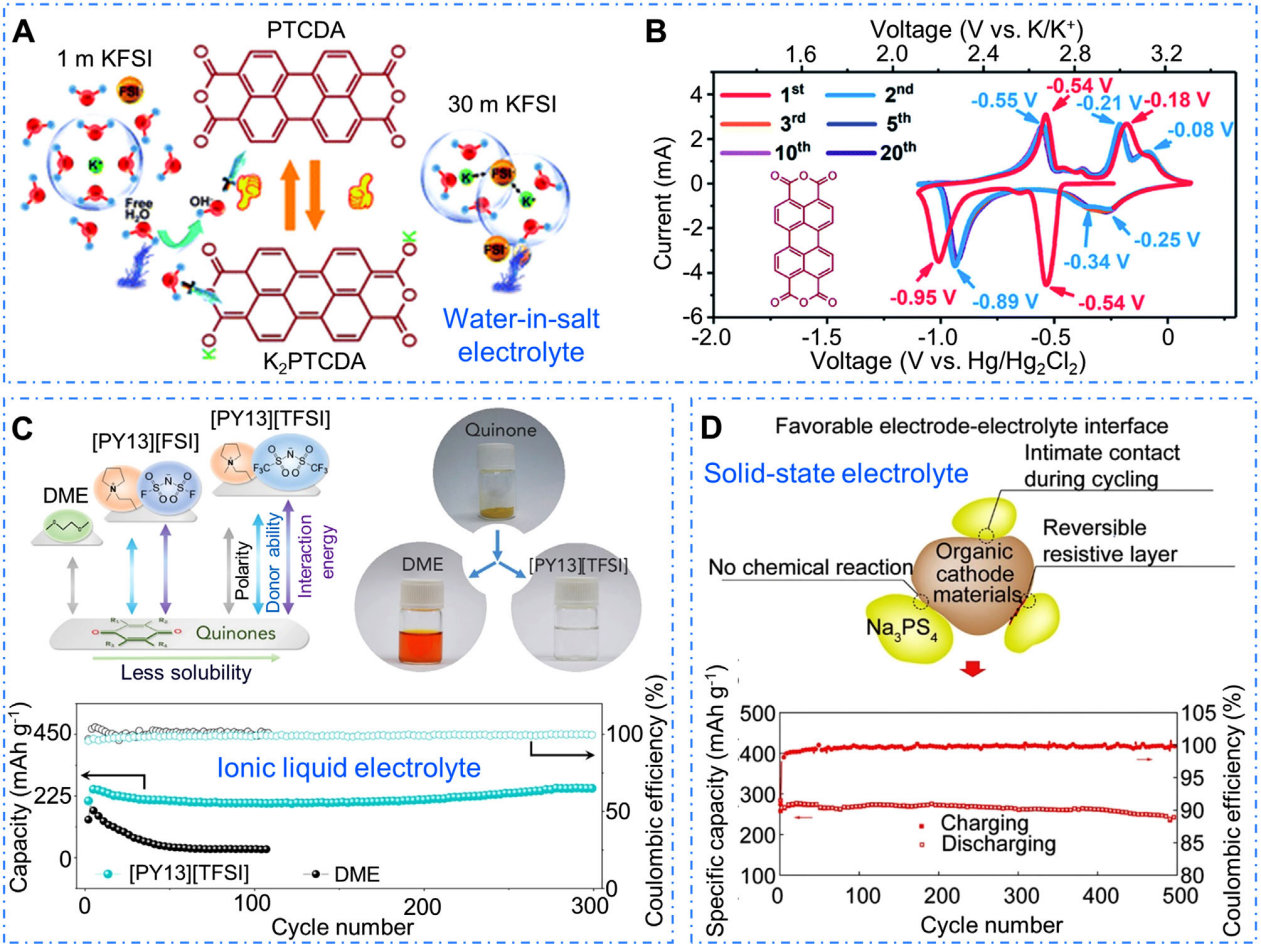
(A) Solvated configurations of PTCDA in 1 and 30 M KFSI electrolyte, respectively. (B) CV curves of PTCDA electrode in a three‐electrode battery at 10 mV s^–1^. Reproduced with permission.^[^ ^107^ ^]^ Copyright 2020, The Royal Society of Chemistry. (C) The solubility of quinones in different solvents and corresponding cycling stability of C4Q cathode. Reproduced with permission.^[^ ^108^ ^]^ Copyright 2019, Elsevier Inc. (D) The potential electrode‐electrolyte interface interaction between PTO cathode materials and Na_3_PS_4_ electrolyte, and the long‐term cycling performance of PTO cathode with solid‐state electrolyte. Reproduced with permission.^[^ ^109^ ^]^ Copyright 2019, Elsevier Inc.

Second, optimizing the electrolyte is an effective method to enhance cycle stability of the overall battery, including regulated electrolyte, ionic liquid electrolyte, solid‐state electrolyte, functional additives, and so on.^[^ ^103^, ^104^, ^105^, ^106^ ^]^ Wang's research group reported a concentrated water‐in‐salt electrolyte to bind free water molecule in aqueous potassium ion batteries (Figure 12A).^[^ ^107^ ^]^ The water‐in‐salt electrolyte with limited free water effectively reduced the solubility of organic materials, thereby significantly increasing the cycling stability of perylene‐3,4,9,10‐tetracarboxylic dianhydride (PTCDA) without hydrogen evolution in Figure 12B. Chen et al. used a kind of ionic liquid (named as [PY13][TFSI]) as the electrolyte to suppress the diffusion of C4Q molecules (Figure 12C), and the C4Q cathode exhibited a capacity retention of ∼99.7% after 300 cycles, which was much better than that in the ester‐based electrolyte.^[^ ^108^ ^]^ Yao's group reported a sulfide‐based solid‐state electrolyte with good electrochemical and mechanical compatibility to the pyrene‐4,5,9,10‐tetraone cathode (PTO). The PTO‐based cell showed a high specific energy (587 Wh kg^–1^) and outstanding cycle stability (a capacity retention of 89% after 500 cycles) in Figure 12D.^[^ ^109^ ^]^ Besides, the introduction of functional additives in the rechargeable metal batteries is mainly blocking the contact between soluble OEMs and metal anode,^[^ ^110^ ^]^ and an example is the usage of an ether‐based electrolyte with LiNO_3_ additive in organotrisulfide‐based lithium batteries.^[^ ^111^ ^]^ With the current collector of binder‐free multi‐walled carbon nanotubes, the dimethyl trisulfide cathode with a high mass loading of 6.7 mg cm^–2^ delivered prolonged cycles in the regulated electrolyte, which was expected to promote the structural engineering and performance exploration of organotrisulfide‐based cathode materials. The electrolyte optimization strategy is useful to stabilize the electrolyte/electrode interfaces, which is beneficial for the enhanced cycling performance of OEMs. The interactions among the ions, redox active sites, and electrolytes make a difference in the overall electrochemical performance of the sustainable batteries, and more systematic researches are needed to comprehensively explore the internal changes and redox mechanism at the battery level.

**FIGURE 13:**
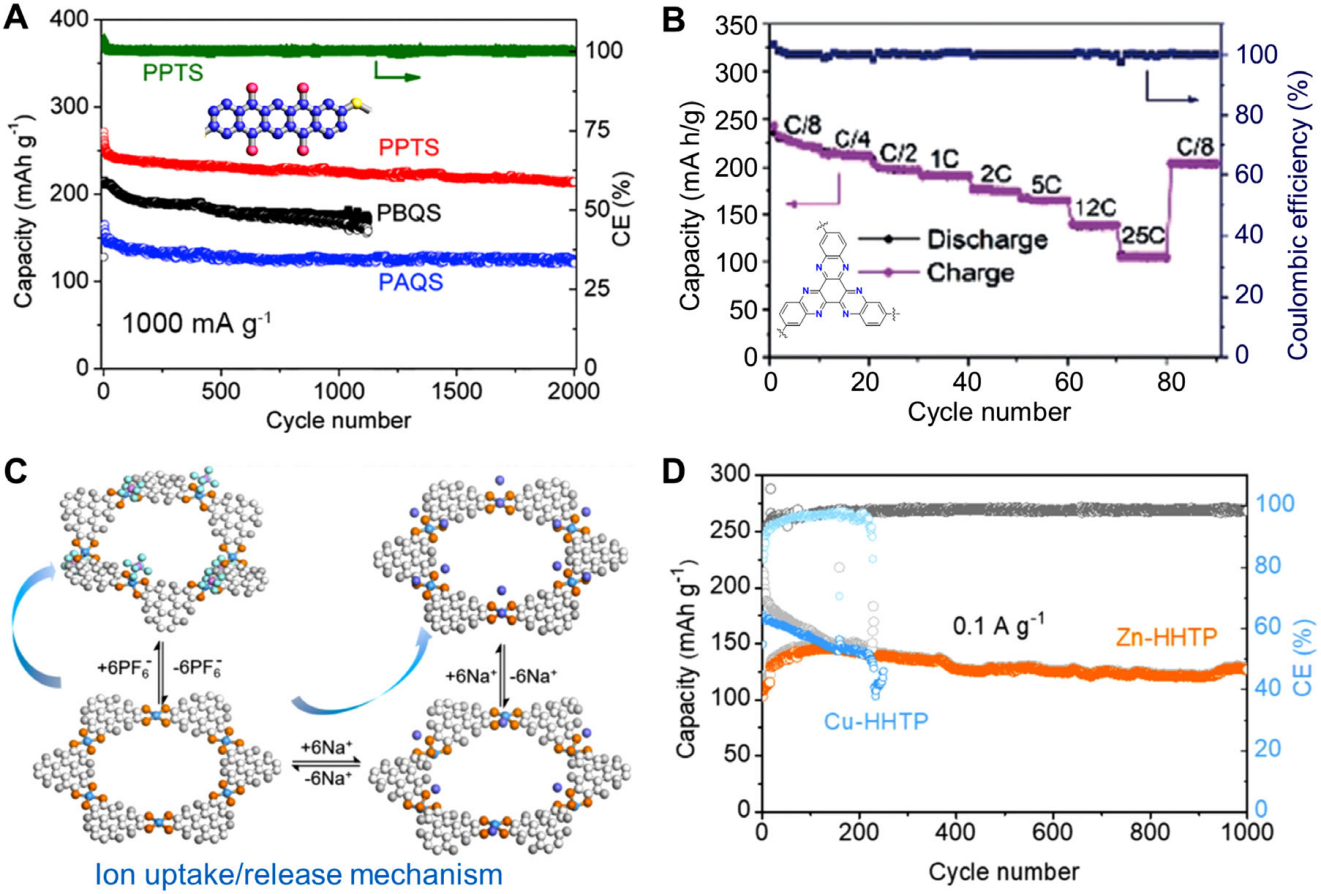
(A) Cycling stability of PPTS, PBQS, and PAQS at a current density of 1 A g^–1^. Reproduced with permission.^[^ ^120^ ^]^ Copyright 2018, Elsevier Inc. (B) The rate capability of PHATN electrode, with a discharge capacity of 100 mAh g^–1^ at the current density of 10 A g^–1^. Reproduced with permission.^[^ ^125^ ^]^ Copyright 2019, WILEY‐VCH GmbH. (C) The redox mechanism and (D) the long‐term cyclability of Zn‐HHTP electrode. Reproduced with permission.^[^ ^130^ ^]^ Copyright 2021, WILEY‐VCH GmbH

Third, polymerization is another effective method to improve the structure stability of organic molecules.^[^ ^112^, ^113^ ^]^ The conductive polymers have attracted the attention of researchers because of their higher electronic conductivity and fast redox reaction kinetics.^[^ ^114^ ^]^ However, the unsatisfying structure stability and low discharge capacity (∼100 mAh g^–1^) limit the rapid development of conductive polymer electrodes in rechargeable batteries.^[^ ^115^ ^]^ Song and Wang have designed some conjugated organic materials such as PBQS, PAQS, P14AQ used as the cathode materials for rechargeable organic batteries.^[^ ^41^, ^116^, ^117^, ^118^ ^]^ P14AQ electrode with stable conjugated structure showed excellent cycling stability (a capacity retention of 99.4% after 1,000 cycles),^[^ ^119^ ^]^ suggesting the effectiveness of the polymerization strategy. Wang et al. reported a rigid conjugated poly(pentacenetetrone sulfide) polymer (PPTS) used as the cathode for organic sodium battery (Figure 13A), and the PPTS electrode showed superior rate capability (∼100 mAh g^–1^ at 50.0 A g^–1^) and a capacity retention of 97% after 10,000 cycles.^[^ ^120^ ^]^ Interestingly, this conjugated polymer with layer‐by‐layer stacking model and intrinsic structure stability provided fast Li^+^ transfer path, enabling the organic battery with excellent rechargeability.

Besides, conjugated microporous polymers (CMPs) emerge as the promising polymer‐based electrode materials owing to their high surface area, structural stability, flexibility, and sustainability.^[^ ^121^, ^122^ ^]^ The application of CMPs in energy storage devices arises rapidly as well, owing to the booming development of COFs recently.^[^ ^123^ ^]^ Chen's group reported a conjugated polymer with carbonyls and imide sites (TQBQ‐COF) used as cathode for the organic sodium battery. The TQBQ‐COF electrode possessed a high discharge capacity and excellent rechargeability (134.3 mAh g^–1^ at 10.0 A g^–1^), which could be attributed to the enhanced heteroatomic doping and rigid nanopores.^[^ ^124^ ^]^ Wang and colleagues employed a pyrazine‐based conjugated polymer (PHATN) as a universal cathode material for rechargeable batteries (Figure 13B), which delivered a capacity of 100 mAh g^–1^ at 10.0 A g^–1^ after 50,000 cycles in sodium battery.^[^ ^125^ ^]^ Chen et al. designed a series of COFs with orthoquinone redox‐active sites used as electrode materials in the supercapacitor devices, and the 4KT‐Tp‐COF‐based electrode with highly reversible redox activity displayed a capacitance of 583 F g^–1^ at 0.2 A g^–1^ and a capacitance retention of ∼92% at 5.0 A g^–1^ after 20,000 cycles.^[^ ^126^ ^]^ It should be noted that CMPs with 2D conjugated structure are featured with ordered pore channel and rigid layered structure, and further pore structure regulation and redox‐active sites utilization are two crucial parameters for improving the electrochemical performance.

Additionally, metal‐organic frameworks (MOFs) with structural versatility, tunable components, and excellent stability are promising electrode materials for future energy storage devices, while the study for MOFs used in battery systems is still in the early stage.^[^ ^127^, ^128^ ^]^ Chen et al. reported a redox‐active 2D conductive MOFs based on the coordination between tetrahydroxy‐1,4‐quinone (THQ) and copper salt, and the Cu‐THQ cathode presented a highly reversible capacity of 387 mAh g^–1^ and good cycling stability.^[^ ^129^ ^]^ Wang et al. constructed π‐d conjugated coordination polymers with triphenylene catecholate (HHTP) and zinc salt.^[^ ^130^ ^]^ As shown in Figure 13C,D, benefiting from the facilitated delocalization of electrons on the conjugated structure, the Zn‐HHTP electrode delivered a reversible capacity of ∼150 mAh g^–1^ and remarkable cycling stability (a capacity retention of 90% after 1,000 cycles). Moreover, Li et al. designed a conductive MOF with redox‐active tricycloquinazoline and CuO_4_ units for lithium storage, and the conductive MOF‐based electrode showed a high specific capacity of 657.6 mAh g^–1^ at 600 mA g^–1^, owing to its multiple redox‐active organic moieties.^[^ ^131^ ^]^ MOFs with redox‐active sites and rigid channel structure are ideal electrode materials, and more effort should be made to find high‐performance MOFs‐based electrode materials and make clear of the ionic insertion/deinsertion process.

**FIGURE 14:**
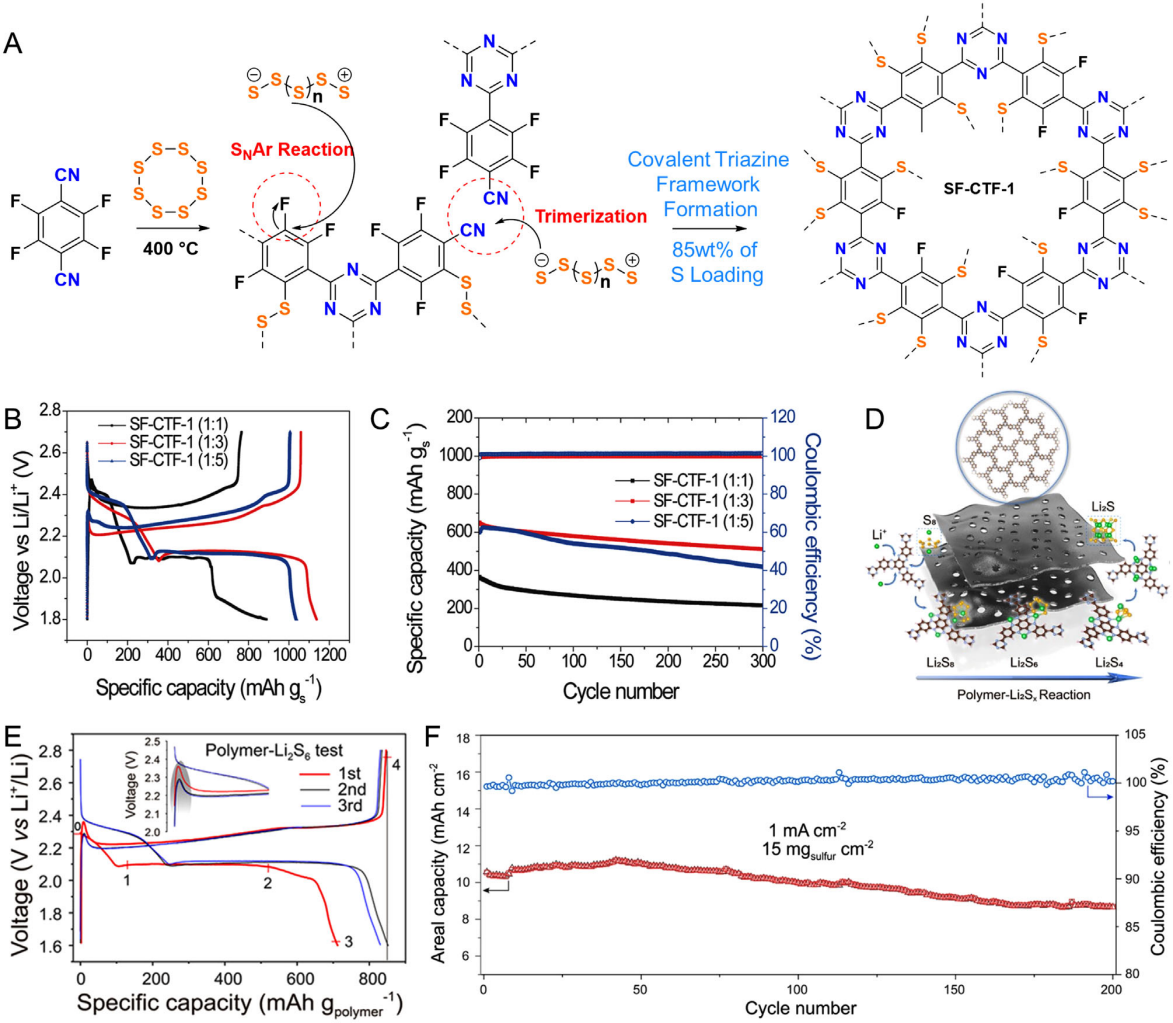
(A) Schematic reaction mechanism and chemical structure of SF‐CTF‐1 composites with high sulfur contents. The first discharge‐charge curves (B) and rate performance (C) of SF‐CTF‐1 cathodes with varying sulfur ratios. Reproduced with permission.^[^ ^132^ ^]^ Copyright 2017, WILEY‐VCH GmbH. (D) The schematic of Li_2_S*_x_*‐reactive pathway of CMPs/S cathode. (E) Voltage profiles of CMPs‐Li_2_S_6_ testing and the first three discharge/charge curves. (F) Long‐term cycling performance of CMPs/S cathode at 1 mA cm^–2^ under an electrolyte/sulfur ratio of 5 μl mg^–1^. Reproduced with permission.^[^ ^133^ ^]^ Copyright 2021, WILEY‐VCH GmbH

Furthermore, CMPs with redox‐active sites are gradually introduced into the Li‐S batteries to anchor polysulfide and accelerate the redox reaction kinetics, which are excepted to remain the merits of ultrahigh energy density (2600 Wh kg^–1^) and sustainability of Li‐S batteries. For example, Choi and colleagues put forward an elemental sulfur‐mediated synthesis coupled with a perfluorinated covalent triazine framework (SF‐CTF‐1) for high sulfur‐loading contents in Figure 14A. The SF‐CTF‐1 composites showed a discharge capacity of 1138.2 mAh g^–1^ at 0.05 C and outstanding cycling stability (a capacity retention of 81.6% after 300 cycles) in Li‐S batteries, as shown in Figure 14B,C.^[^ ^132^ ^]^ Besides, Loh et al. developed a densely stacked and redox‐active CMPs as the sulfur host in Figure 14D.^[^ ^133^ ^]^ Benefiting from the enhanced binding polysulfide interaction and fast sulfur transformation chemistry within the bulky polymer carrier, the CMPs/sulfur cathode exhibited a high areal capacity of ∼14 mAh cm^–2^ and a cell‐level high energy density of 303 Wh kg^–1^ (Figure 14E,F). As a result, CMPs with structural designability and functional diversity are promising alternatives to traditional OEMs in rechargeable batteries, and the exploration of novel OEMs and energy storage devices is still a crucial part for the application of rechargeable organic batteries.^[^ ^134^ ^]^

Notably, there is a booming development of OEMs (such as COFs and MOFs) used as electrolyte materials to avoid the continuous loss of active materials and improve the compatibility between electrode and electrolyte, owing to the merits of bountiful resources, structural designability, and sustainability of OEMs. The traditional organic polymer‐based electrolytes suffer from the poor ionic conductivity and limited interface stability. To enhance the ionic conductivity and structural stability of COF‐based solid‐state electrolyte, Niu et al. prepared flexible COF electrolyte films with lithiophilic groups and quinolyl aromatic ring linkages in the solid‐state LIBs.^[^ ^135^ ^]^ The COF‐based solid‐state electrolyte films showed rapid Li^+^ transfer (1.5 × 10^−4^ S cm^–1^ at 60°C) by directional hopping paths in rigid nanopores of COF, and the assemble all‐solid state LIBs maintained the long‐term cycling over 400 cycles. Manthiram and co‐workers presented an electrolyte‐mediated single‐Li^+^ conductive COF with in situ polymerized dimethylacrylamide (DMA) for foldable solid‐state batteries.^[^ ^136^ ^]^ The DMA@LiTFSI mediated COF electrolyte was endowed with high charge‐carrier concentration in the COF channels, accelerating the movement of Li^+^ in an ordered manner (1.7 × 10^−4^ S cm^−1^ at RT) among the flexible polymer chains and rigid COF backbone. Besides, Zhou's research group reported a MOF‐based quasi‐solid electrolyte with inside channels of 6.5 Å, demonstrating its advantages in weight and safety when applied in a high‐voltage LiNi_0.8_Co_0.1_Mn_0.1_O_2_//Li pouch cell.^[^ ^137^ ^]^ MOF quasi‐solid electrolyte with poly(sodium 4‐styrenesulfonate) decorated inside its channels delivered a capacity of 164 mAh g^−1^ after 100 cycles even under harsh conditions. Consequently, OEMs with functional groups, structural designability, and degradation could be used as versatile electrode materials, offering an attractive solution to develop the high‐stable and eco‐friendly batteries.

#### 3.2.4 Strategies to improve the electronic conductivity of OEMs

**FIGURE 15:**
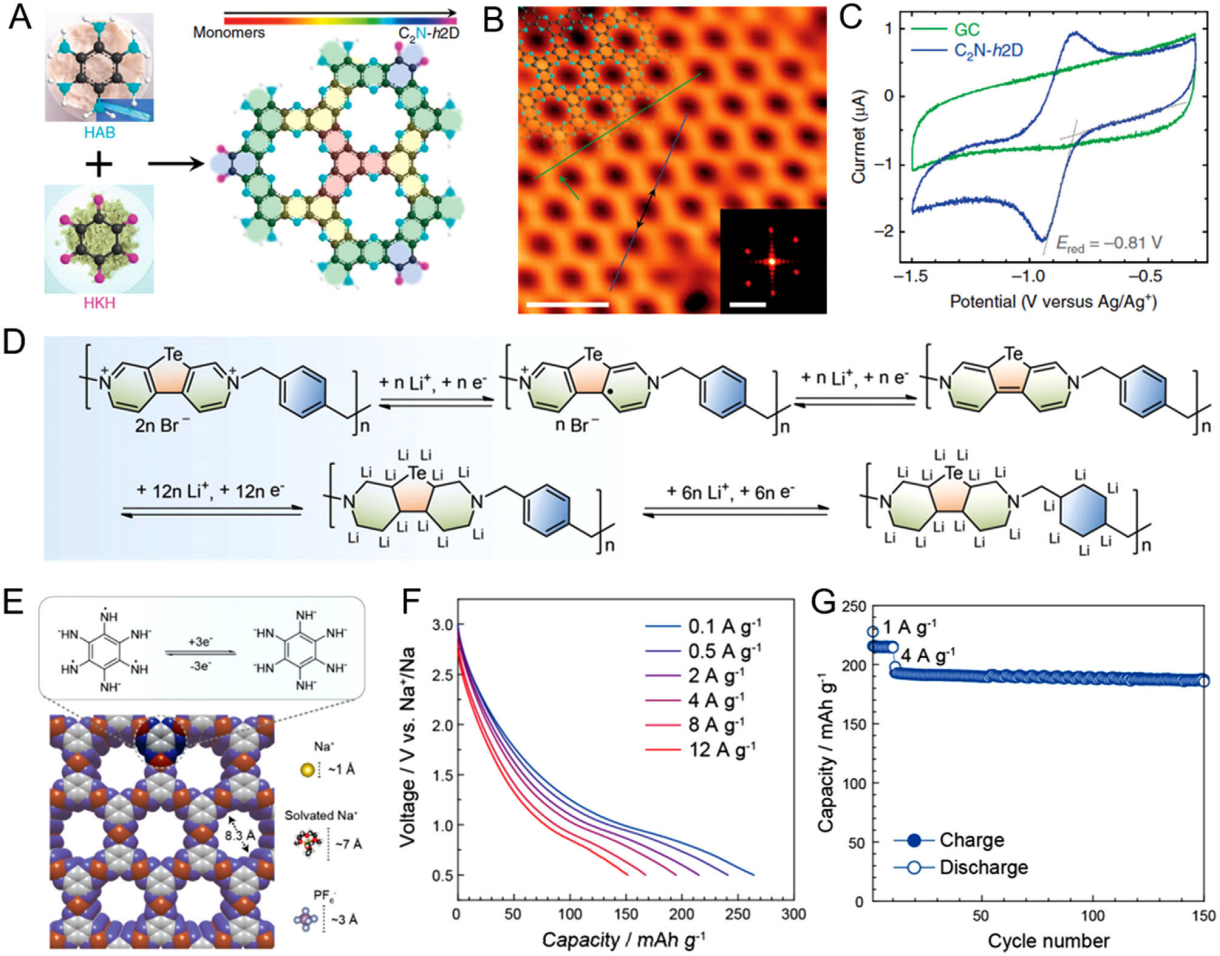
(A) Schematic synthesis route of C_2_N‐*h*2D crystal. (B) Scanning tunneling microscope image on Cu (111) and (C) CV curve at 0.1 mV s^–1^ of the C_2_N‐*h*2D crystal. Reproduced with permission.^[^ ^147^ ^]^ Copyright 2015, Nature Publishing Group. (D) Proposed Li^+^ storage mechanism of the Te‐based viologen scaffold electrode. Reproduced with permission.^[^ ^154^ ^]^ Copyright 2019, WILEY‐VCH GmbH. (E) The simulated structure of Co‐HAB with three‐electron reversible reaction. (F) Discharge profiles of Co‐HAB electrode at different current densities. (G) Cycling stability of Co‐HAB electrode. Reproduced with permission.^[^ ^155^ ^]^ Copyright 2018, The American Chemical Society

Based on the energy‐band theory, almost all of the organic compounds, except for conducting polymers, are almost electrical insulators (∼10^−15^ S cm^–1^) with a large energy‐band difference between conduction band and valence band.^[^ ^138^, ^139^, ^140^ ^]^ OEMs with poor electronic conductivity always show low utilization of active materials and inferior rate capability of the overall batteries, hindering the wide application of OEMs.^[^ ^141^ ^]^ Conducting polymers (such as polypyrrole, polythiophene, polyaniline) show an enhanced electronic conductivity through the n‐doping/p‐doping strategy, which is within the range of the electronic conductors.^[^ ^142^ ^]^ Notably, conducting polymer electrodes are limited by their low theoretical capacity and complex redox mechanism, while conducting polymers with excellent flexibility could be used to modify the inorganic electrodes to improve their cycling stability. Moreover, heteroatomic (i.e., N, O, S, B) doping is usually used to reduce the energy‐band difference and improve the electronic conductivity of organic compounds, especially for the conjugated organic polymers.^[^ ^143^, ^144^, ^145^ ^]^ For example, HATN is employed with N‐rich sp^2^ hybridization structure, giving a band gap of 4.73 eV between the LUMO and the highest occupied molecular orbital.^[^ ^146^ ^]^ As shown in Figure 15A–C, Baek et al. reported a layered nitrogenated holey crystal, with experimental bandgaps of about 1.96 eV, which was comparable to that of the semiconductor.^[^ ^147^ ^]^ Feng et al. reported a HATN‐based conjugated polymer with the sp^2^ hybridization structure, and it showed an intrinsic electronic conductivity of 9.1 × 10^−10^ S cm^–1^ and 53% utilization of the redox‐active pyrazine sites.^[^ ^148^ ^]^ However, the heteroatomic doping method does limited effect on improving the electronic conductivity of OEMs.

Moreover, some organic salts exhibit a high electronic conductivity, such as the oxocarbon salts (2.5 × 10^−5^ S cm^–1^) and polyazaacene es (1.0 × 10^−5^ S cm^–1^), one order of magnitude lower than those of the inorganic materials (∼10^−4^ S cm^–1^ for LiCoO_2_).^[^ ^149^, ^150^ ^]^ Alternatively, introducing delocalized π‐electron systems or inorganic into the skeleton structure of the polymers via the function‐oriented molecular design strategy is another good solution to promote the charge‐transfer process as well.^[^ ^151^, ^152^, ^153^ ^]^ For example, He's research group introduced the chalcogen atoms (S, Se, and Te) into the viologen scaffold in Figure 15D, thus contributing the improved the electronic conductivity of Te‐poly(chalcogenoviologen)s polymers to 1.3 × 10^−6^ S cm^–1^.^[^ ^154^ ^]^ However, the current organic electrodes with hetero atoms are usually showing poor electronic conductivity, and the doping strategy has certain limitations in effectively accelerating the electron transfer for the individual compounds. Besides, due to the enabled delocalization of electrons in the whole π‐d conjugated structure, MOFs with π‐d hybrid orbitals from metal ions and organic ligands also exhibit enhanced electrical conductivity. Bao and colleagues reported a 2D conductive MOFs based on the coordination between hexaaminobenzene (HAB) and cobalt salt as shown in Figure 15E.^[^ ^155^ ^]^ The Co‐HAB electrode presented an electronic conductivity of 1.57 S cm^–1^ and good electrochemical performance (Figure 15F,G). Although this strategy increases the electrical conductivity of OEMs to a higher level, their actual capacities are affected by the large parts of inactive ingredients. How to design the structure with high electronic conductivity and high capacity will be an important topic for a long time for OEMs.

**FIGURE 16:**
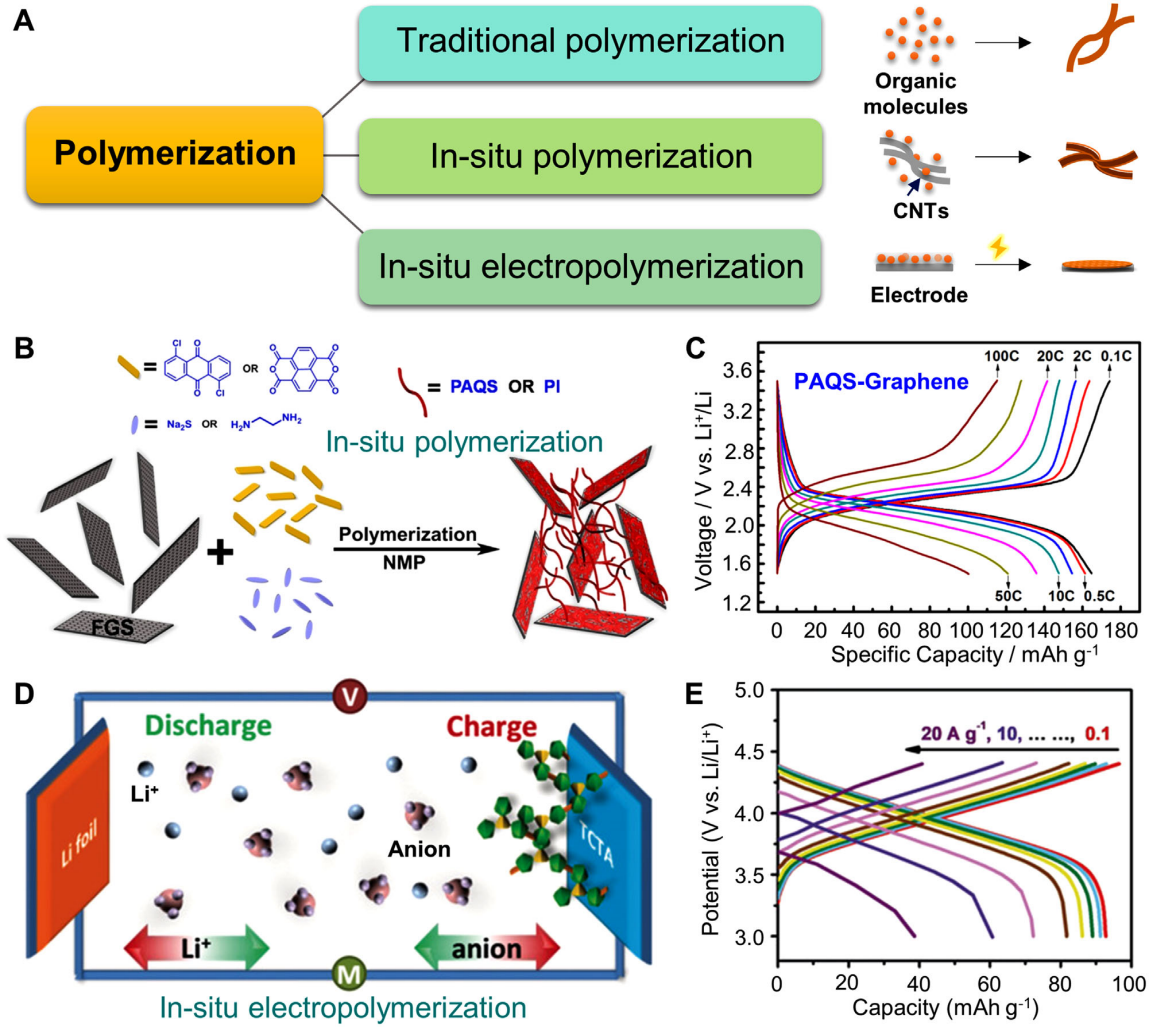
(A) The polymerization types for connecting small organic molecules. (B) The in situ polymerization process and schematic diagram of PAQS‐GO composites. (C) The rate capability of PAQS‐GO electrode at the current density from 0.1 to 100 C. Reproduced with permission.^[^ ^160^ ^]^ Copyright 2012, The American Chemical Society. (D) The in situ electropolymerization illustration and schematic redox chemistry mechanism of carbazole‐based cathode for organic lithium batteries. (E) The rate performance of carbazole‐based cathode during the current density of 0.1–20.0 A g^–1^. Reproduced with permission.^[^ ^162^ ^]^ Copyright 2020, WILEY‐VCH GmbH

Additionally, the low electronic conductivity of OEMs could be efficiently improved by introducing the conductive additives (10–60%).^[^ ^156^ ^]^ Within a certain amount of carbon additive, the structure and morphology engineering for the organic electrodes is necessary to improve the utilization of the active materials without affecting the mass energy density.^[^ ^157^, ^158^ ^]^ Based on this consideration, polymerization with conductive carbon (Figure 16A) is an effective method to adjust the electron transport behavior.^[^ ^159^ ^]^ Wang and colleagues designed polymer‐graphene nanocomposites by in situ polymerization method in Figure 16B.^[^ ^160^ ^]^ The nanocomposite electrodes with enhanced electronic conductivity (6.4 × 10^−3^ S cm^–1^) and utilization of the carbonyl groups could show a discharge capacity of approximately 100 mAh g^–1^ within just 16 s (Figure 16C). Abruña and co‐workers constructed a PZ‐based COF electrode with high Li^+^ diffusion capability, and its composite with conductive poly(3,4‐ethylene dioxythiophene) (PEDOT) exhibited an electronic conductivity of 7.2 × 10^−5^ S cm^–1^, boosting its improved electrochemical accessibility of redox‐active sites.^[^ ^161^ ^]^ Xu et al. developed an in situ electrochemical polymerization to stabilize the nanosheet structure and improve the electronic conductivity of the carbazole‐based cathodes (Figure 16D), and the electro‐polymerized electrode demonstrated a high discharge voltage of 3.95 V and ultrafast rate performance at 20.0 A g^–1^ in Figure 16E.^[^ ^162^ ^]^ This work adopts a novel strategy of the in situ electro‐polymerization method to fabricate the organic electrode, and the severe aggregation behavior and unstable contact of the active materials and conductive carbon are effectively avoided. Nevertheless, the introduction of inactive groups and components decreases the theoretical specific capacity of OEMs.^[^ ^163^, ^164^ ^]^ Designing OEMs with high content of active ingredients and reversible redox activity, combined with targeted strategies, is an efficient method to promote the broad application of organic materials in next‐generation rechargeable batteries.

#### 3.2.5 Strategies to confirm the complex redox mechanism of OEMs

**FIGURE 17:**
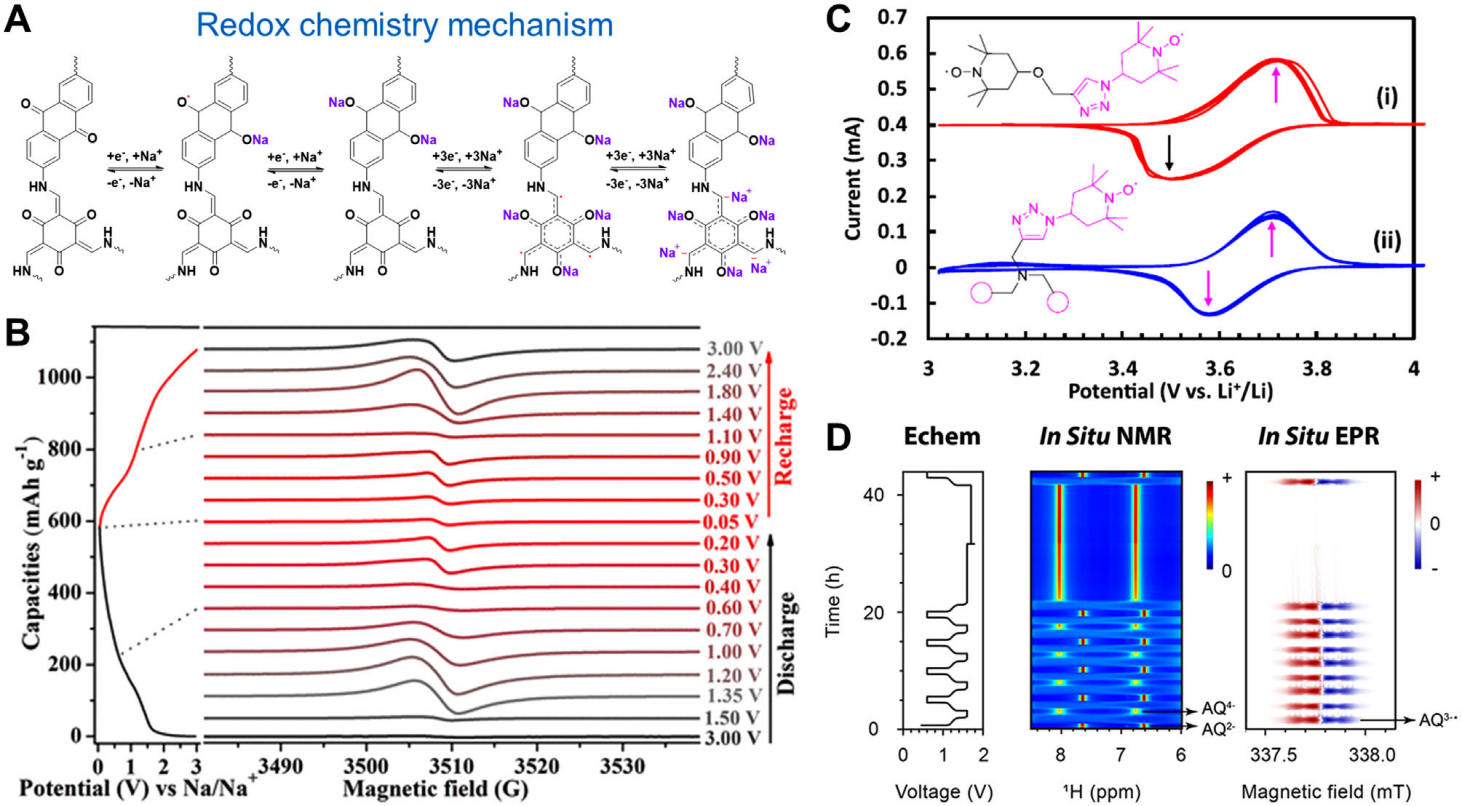
(A) Electrochemical redox reaction mechanism and (B) EPR spectra change of radical intermediates during the sodium storage process. Reproduced with permission.^[^ ^165^ ^]^ Copyright 2019, The American Chemical Society. (C) CV curves of organic electrode materials consist of TEMPO structure at the scan rate of 2.0 mV s^–1^. Reproduced with permission.^[^ ^166^ ^]^ Copyright 2021, Elsevier Inc. (D) The coupled EPR/NMR methods for determining redox‐active DHAQ intermediates during electrochemical cycling process. Reproduced under the terms of the CC‐BY license.^[^ ^167^ ^]^ Copyright 2021, The American Chemical Society

The radical intermediates widely exist in the conductive polymers, and researchers have realized the existing radical intermediates in most organic electrodes with the aid of the electron paramagnetic resonance (EPR) spectrum. Lu et al. revealed that the state of radical intermediates (including C‐O^•^ and α‐C) by EPR spectra in Figure 17A,B, which were essential to the cycle stability and contribution capacity of the COFs‐based electrode.^[^ ^165^ ^]^ Nitroxide radical polymers, including TEMPO radicals (Figure 17C), attracted more and more attention for their high structural stability and rapid redox kinetics, which are widely applied in the catalytic and energy storage areas as well.^[^ ^166^ ^]^ As shown in Figure 17D, Grey et al. investigated the radical anion of 2,6‐dihydroxyanthraquionone (DHAQ^3–•^) as a reaction intermediate during electrochemical cycling of redox flow batteries, determining the electron transfer parameters between DHAQ^3–•^ and DHAQ^4–^ anions by the coupled EPR and nuclear magnetic resonance (NMR) methods.^[^ ^167^ ^]^ To elucidate the Na^+^ ions uptake/release process of PPTO polymers, Chen et al. investigated the most possible existing states of the radical intermediates by the DFT calculations and ERR spectra, respectively, confirming the four Na^+^ redox chemistry mechanisms of per PPTO unit.^[^ ^168^ ^]^ Therefore, further study on the intermediates tunability of organic electrodes is an indispensable part of the enhanced electrochemical performance of rechargeable secondary batteries.

**FIGURE 18:**
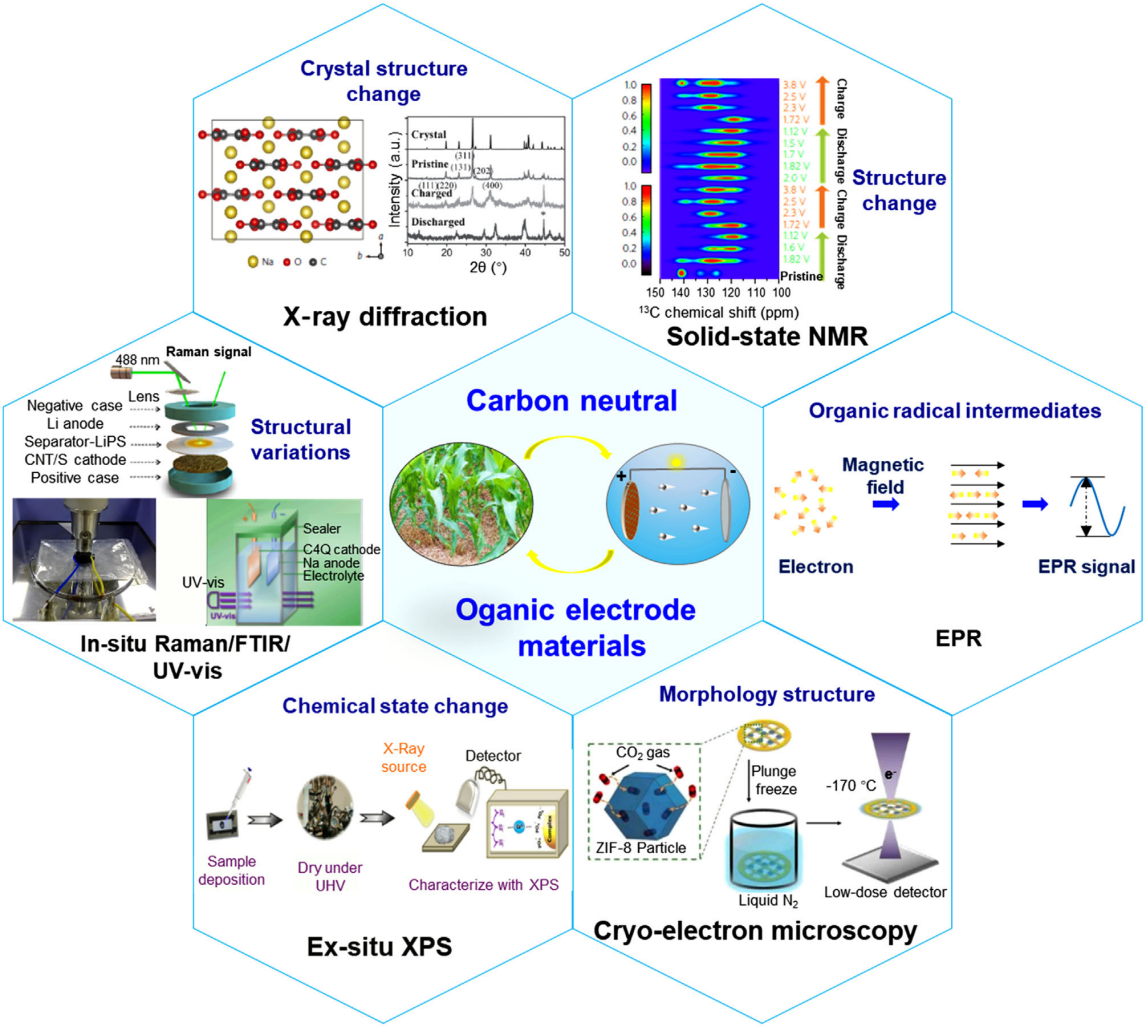
Advanced characterization techniques for real‐time structural and composite changes of OEMs: X‐ray diffraction, solid‐state NMR, in situ FTIR, in situ Raman, in situ UV–vis, EPR, ex situ XPS, Cryo‐electron microscopy. Reproduced with permission from the reported references.^[^ ^75^, ^102^, ^108^, ^171^, ^176^, ^177^, ^178^, ^179^ ^]^ Copyright 2017, Nature Publishing Group. Copyright 2019, Elsevier Inc. Copyright 2018 and 2016, WILEY‐VCH GmbH. Copyright 2017 and 2021, The American Chemical Society

Advanced characterization methods: An in‐depth study on the redox reaction mechanism cannot be carried out without the help of advanced characterization methods.^[^ ^169^ ^]^ OEMs with redox‐active sites are usually manifested by the Fourier transform infrared spectroscopy (FTIR), NMR spectrum, UV–visible spectroscopy, Raman spectra, X‐ray photoelectron spectroscopy, and so on.^[^ ^108^, ^170^, ^171^ ^]^ In/Ex situ FTIR spectra are widely used to test the structural evolution of the reactive functional groups of organic electrodes accompanying a multiple electron transfer process (Figure 18). Zhao et al. reported a series of quinone electrodes (C4Q in particular) coupled with the Nafion membrane in the pouch‐type zinc batteries, exhibiting an energy density of 220 Wh kg^–1^ based on the total mass of C4Q and theoretical Zn anode.^[^ ^43^ ^]^ The solubility and structural changes of quinone electrode materials were investigated by in situ spectral techniques including FTIR, Raman, and UV–visible spectra.^[^ ^172^ ^]^ Loh et al. constructed a HATN‐based electrode with 60 wt% graphene,^[^ ^75^ ^]^ and ex situ solid‐state ^15^N NMR and ^13^C NMR spectra were applied to elucidate the structural evolution and multiple lithiation mechanism of HATN cathode cycled to different states. Kang's group reported a MOF‐gel separator with molecular and ionic sieving capability for the rechargeable organic batteries, and hence the 5,5′‐dimethyl‐2,2′‐*bis*‐p‐benzoquinone (Me_2_BBQ) cathode displayed a high capacity retention of 82.9% after 2,000 cycles.^[^ ^100^ ^]^ The dissolution behavior of Me_2_BBQ cathode during the lithiation/delithiation process was monitored by the UV–vis spectra, revealing the solid‐liquid‐solid conversion during the electrochemical reaction.

Additionally, some organic salts and rigid COFs can be further confirmed by the powder X‐ray diffraction (XRD) spectrum and the selected area electron diffraction image from high‐solution electron microscopy.^[^ ^173^ ^]^ About a decade later, Bao's research group made it clear that the irreversible phase transformation of Na_2_C_6_O_6_ during cycling is the origin of the deteriorating electrochemical performance, by means of the operando XRD data.^[^ ^102^ ^]^ Wang et al. constructed a two‐dimensional COFs‐based anode coupled with CNTs for LIBs, and the 14‐electron redox chemistry mechanism for per COF monomer was demonstrated by the theoretical calculations and experimental tests (HRTEM, Raman, XPS, etc.) of electrochemical behavior at different cycled states.^[^ ^174^, ^175^, ^176^ ^]^ The advanced operando characterization technologies (such as STM, HRTEM, XPS, FTIR, UV–visible, EPR) need to be developed and applied to elucidate the structure and morphology change of OEMs during the battery operation process.^[^ ^177^, ^178^, ^179^ ^]^

**FIGURE 19:**
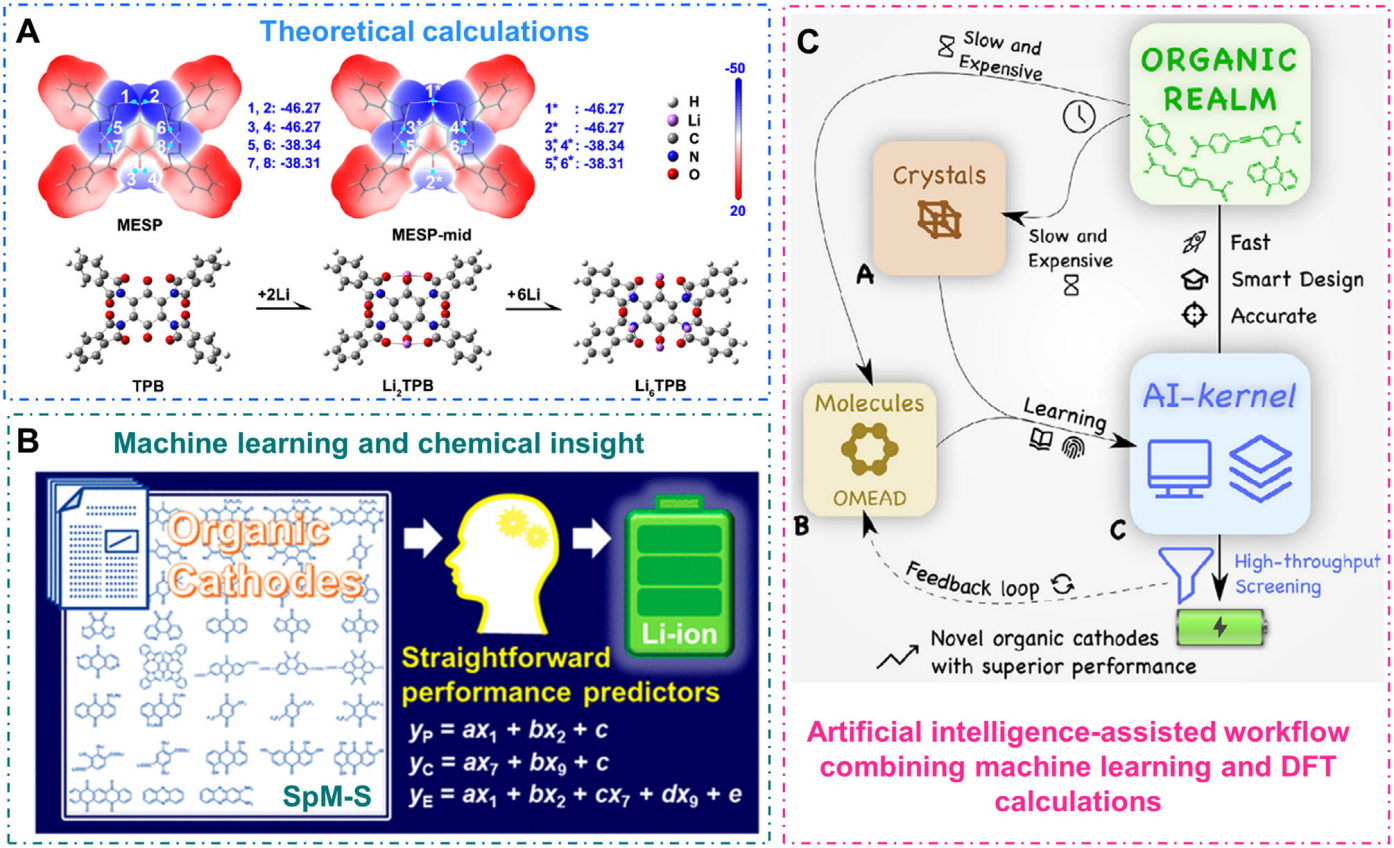
(A) MESP plots and optimized configurations of TPB molecule and its lithiated structures. Reproduced with permission.^[^ ^188^ ^]^ Copyright 2018, The American Chemical Society. (B) Flowchart of the straightforward prediction models to explore high‐performance OEMs. Reproduced with permission.^[^ ^199^ ^]^ Copyright 2022, The American Chemical Society. (C) The workflow of the artificial intelligence‐based method enabled high‐throughput screening and the efficient searching for novel organic materials. Reproduced with permission.^[^ ^200^ ^]^ Copyright 2022, Elsevier Inc.

Computational techniques: The large possible structural variations and complex interrelation among the various intermediates of OEMs are still challenging to explore thoroughly with experimental techniques.^[^ ^180^, ^181^ ^]^ Particularly, the theoretical calculations have a great potential to understand the redox chemistry mechanism during the charge/discharge process at the molecular state, so as to identify the possible ion binding sites, predict the redox potentials, and simulate the lithiated structures.^[^ ^182^, ^183^ ^]^ The early mathematical models are carried out by predicting corresponding energy of possible intermediate structures, achieving the most favorable ion binding site ultimately.^[^ ^184^ ^]^ During the last decades, proper understanding of the relationship between mathematical models and rapidly changing properties of experimental investigations has promoted the discovery and designability of new materials/combinations thereof.^[^ ^185^, ^186^ ^]^ For example, Jang and colleagues revealed the most likely favored Li^+^ binding positions in 2,6‐diaminoanthraquinone after optimizing five potential redox‐active sites by the DFT calculations, but this conventional modeling process was time‐consuming and imprecise.^[^ ^187^ ^]^ Combined with the DFT calculations, Chen et al. developed the molecular electrostatic potential (MESP) method to calculate the accurate Li^+^ binding sites of tetra‐(phthalimido)‐benzoquinone (TPB) molecule. MESP method predicted the lithiated structures and reaction mechanism of redox‐active organic materials more precisely by displaying clear visualizable surface minima points on the simulated TPB structure (Figure 19A).^[^ ^188^ ^]^ Concerning the complex multiple electron transfer process, researchers continuously introduced more reliable theoretical computational simulation tools to predict optimizing transition‐state structure, redox potentials, and migration pathways in organic batteries.^[^ ^189^, ^190^, ^191^ ^]^

The theoretical calculation methods (DFT calculations, molecular dynamics simulations, transition‐state theory, etc.), describing material structure changes and ion migration pathways, have been considerably developed to predict the battery operation principles at various lengthscale and timescale.^[^ ^185^, ^192^ ^]^ Molecular dynamics simulations are usually applied in complimentary to DFT calculations to model more complex and disorganized organic storage systems.^[^ ^193^, ^194^ ^]^ Oguchi's group used the first‐principles calculations coupled with an evolutionary algorithm to predict the electronic structure of the intermolecule bonding and antibonding states of C_6_O_6_, demonstrating the charge/discharge mechanism of the Na*_x_* C_6_O_6_ cathode in organic sodium batteries.^[^ ^195^ ^]^ Similarly, Manzhos et al. presented atomistic‐scale simulations to calculate the increased voltage of polyaniline with the introduction of cyano groups, combined with molecular dynamics simulations and density functional tight binding (DFTB) methods.^[^ ^196^ ^]^ The theoretical calculations could foresee the structural variations and valence states of intermediates during ionic insertion/deinsertion process of OEMs, and the interconnecting first‐principles computational methodologies with force‐field methods are promising to fasten the data‐driven exploration process in the future energy storage devices.

Moreover, the emerging machine learning technique is a high‐throughput method to explore the valuable information from both experimental and theoretical datasets.^[^ ^197^, ^198^ ^]^ Oaki's research group constructed straightforward prediction models with combination of machine learning and chemical insight (Figure 19B), and the prediction models were efficient to explore high‐performance OEMs in a certain search space by the sparse modeling for small data (SpM‐S).^[^ ^199^ ^]^ Araujo et al. developed an artificial intelligence (AI)‐assisted framework in combination with DFT calculations and machine learning for efficient discovery of novel OEMs (Figure 19C), and the AI‐based workflow achieved high‐throughput screening of 20 million molecules to efficiently search for new organic cathodes candidates.^[^ ^200^ ^]^ The AI‐based workflow is a useful tool for accurately identifying the functionalities of organic molecules without time‐confusing experiments, which will pave the way for the high‐efficient data‐driven exploration and structure design of future electroactive OEMs. Notably, the artificial intelligence‐based machine learning methods could accelerate the discovery process of novel organic electroactive materials and the mechanism analysis of rechargeable batteries as well.^[^ ^201^, ^202^ ^]^ Both experimental and computational techniques are crucial for predicting the properties of redox active organic materials, and the developments in high‐throughput computational methods will promote the rapid application of machine learning in high‐performance organic electrode screening.

## 4 FULL BATTERY DESIGN BASED ON OEMS

Compared with the traditional LIBs, OEMs‐based full batteries are usually constructed with a p‐type organic electrode and a common n‐type organic electrode.^[^ ^203^, ^204^ ^]^ Generally, the overall mass energy density of full organic battery is closely related to the kinds of electrode materials, the ratio of anode and cathode materials, the type and amount of electrolyte.^[^ ^205^ ^]^

### 4.1 All‐organic full batteries

**FIGURE 20:**
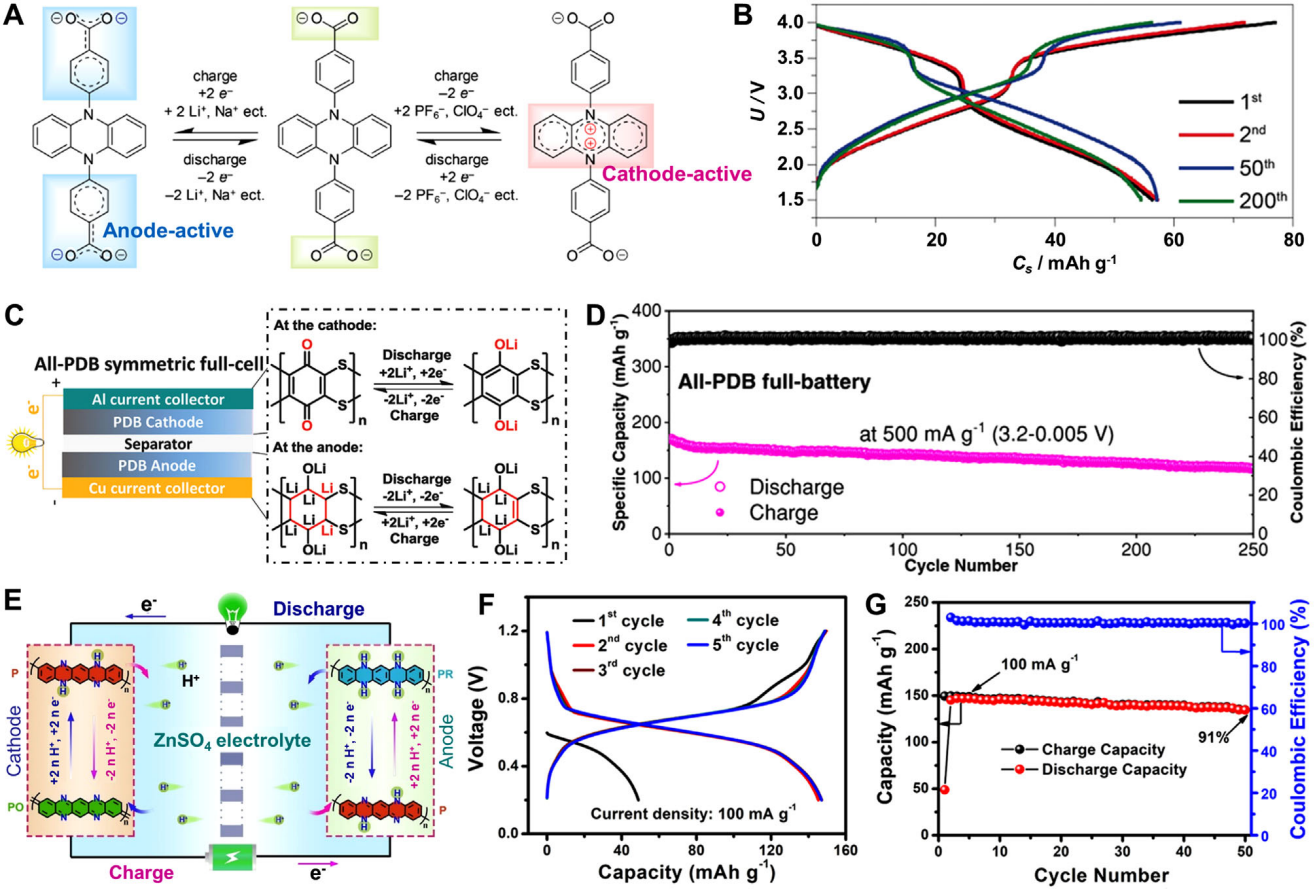
(A) Redox chemistry mechanism of phenazine‐based bipolar electrode and (B) the charge/discharge profiles of organic symmetric battery. Reproduced with permission.^[^ ^207^ ^]^ Copyright 2019, WILEY‐VCH GmbH. (C) Li^+^ ion storage mechanism per electrode of the all‐PDB symmetric full‐cell. (D) The long‐term cycling performance of the all‐PDB symmetric full‐cell. Reproduced with permission.^[^ ^208^ ^]^ Copyright 2018, WILEY‐VCH GmbH. (E) The H^+^ uptake/release mechanism of the symmetric all‐organic proton battery. (F) The discharge/charge curves and (G) cycling performance of the all‐organic proton battery. Reproduced with permission.^[^ ^209^ ^]^ Copyright 2022, WILEY‐VCH GmbH

The all‐organic full batteries are widely studied at the lab level. Chen's research group prepared a series of oxocarbon salts through one‐pot proton exchange reactions for fast rechargeable batteries. Especially, owing to the rapid K^+^ diffusion process, K_2_C_6_O_6_ electrode showed a discharge capacity of 212 mAh g^–1^ and ultrafast rate capacity.^[^ ^206^ ^]^ Zhou et al. designed a PZ‐based bipolar electrode for dual‐ion organic symmetric battery in Figure 20A, which exhibited an energy density of 127 Wh kg^–1^ and almost no capacity decay within 200 cycles at 1 C, suggesting the prospect of OEMs used in the all‐organic symmetric batteries (Figure 20B).^[^ ^207^ ^]^ Then, Zhang et al. reported an all‐plastic full battery with bi‐functional ladderized heterocyclic poly(quinone) (PDB) electrode in Figure 20C, and the symmetric full battery delivered a large capacity of 249 mAh g^–1^ and a capacity retention of 78.3% of after 250 cycles (Figure 20D).^[^ ^208^ ^]^ Recently, Niu's research group prepared a conjugated poly(2,9‐dihydroquinoxalino‐[2,3‐b]phenazine) (PO) molecule as both the cathode and anode for an all‐organic proton battery (Figure 20E).^[^ ^209^ ^]^ As shown in Figure 20F,G, the full proton battery showed a high reversible capacity of 147 mAh g^–1^ and good cycling stability.

Furthermore, OEMs‐based redox flow cells are achieving increasing attention for the booming development of large‐scale energy storage. Jin's research group designed the all‐polymer particle slurry batteries with commercial dialysis membranes, which could promote the rapid H^+^ transfer and inhibit the shuttle effect of redox‐active materials.^[^ ^210^ ^]^ The all‐polymer (polyhydroquinone/polyimide) redox flow cell delivered a reversible capacity of 4.95 Ah L^–1^ and good long‐term cycling (with 70% of capacity retention after 300 cycles). The study for all‐organic full batteries further promotes the exploration and applications of OEMs, paving the way toward the rapid development of sustainable energy storage devices.

### 4.2 OEMs‐based hybrid full batteries

**FIGURE 21:**
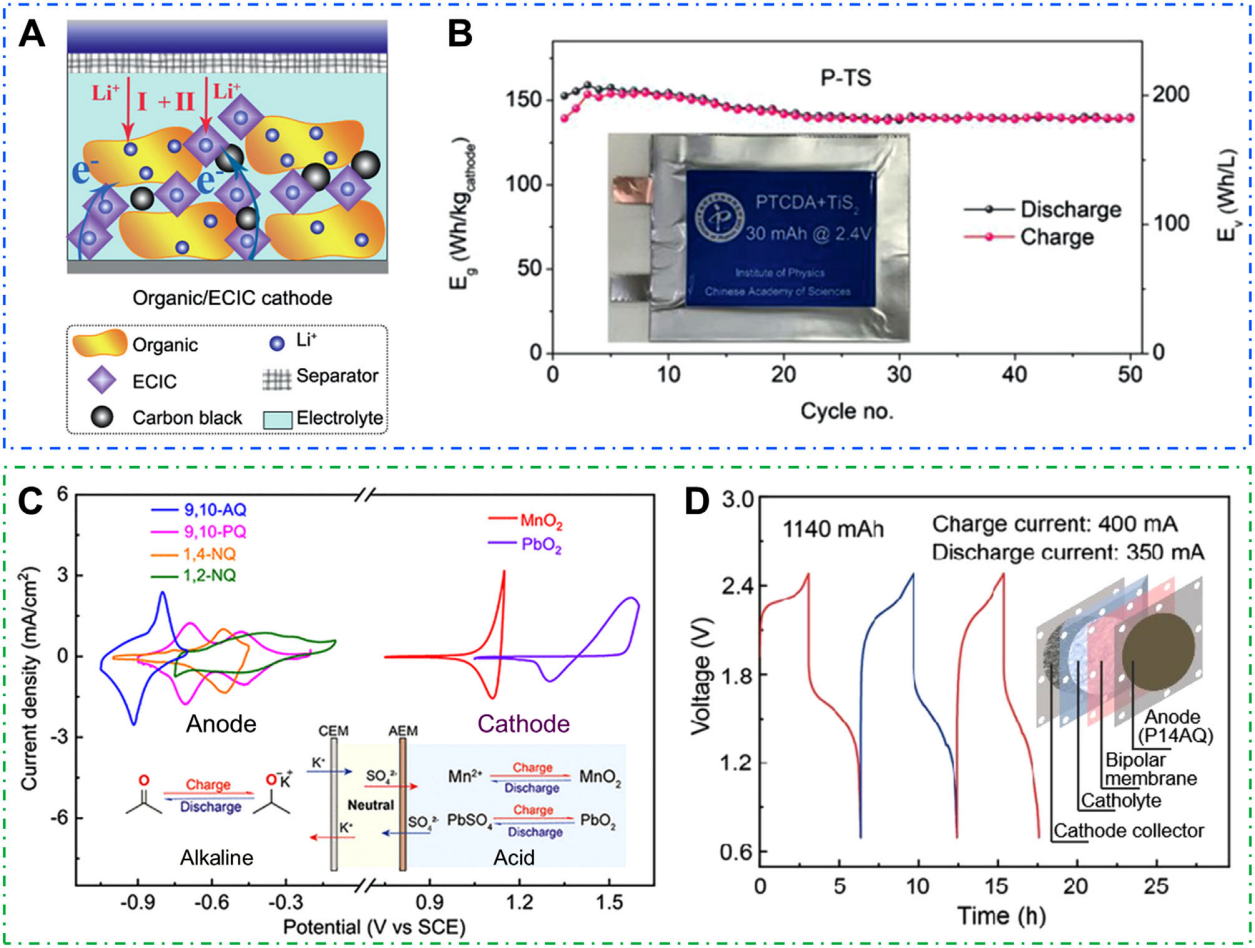
(A) Illustration of the pathways of electrons and Li ions in the inorganic/organic cathode. (B) Cycling performance of 30 mAh‐level Li/PTCDA‐TiS_2_ pouch cell. Reproduced with permission.^[^ ^211^ ^]^ Copyright 2021, WILEY‐VCH GmbH. (C) CV curves and redox reactions of the electrochemical redox couples in the alkali−acid hybrid batteries. (D) Voltage profiles and the schematic structure of P14AQ/MnO_2_ hybrid battery with a high capacity of 33 mAh. Reproduced with permission.^[^ ^212^ ^]^ Copyright 2022, The American Chemical Society

In addition, in view of higher operation voltage platform of inorganic cathodes (such as PbO_2_, LiMn_2_O_4_, LiFePO_4_, NaVPO_4_F), researchers could also combine the inorganic cathode with n‐type organic anode to broaden the operation voltage window and improve the whole mass energy density of full batteries. For example, Suo and colleagues replaced the inactive carbon with electronic conductive inorganic cathode materials to improve the electronic conductivity of organic‐inorganic hybrid cathode (Figure 21A).^[^ ^211^ ^]^ The combination of inorganic materials (TiS_2_ or Mo_6_S_8_) with OEMs (PTCDA or HATN) endowed the whole OEMs‐based battery with high energy density, and a 30 mAh‐level Li/PTCDA‐TiS_2_ pouch cell displayed a high gravimetric energy of 153 Wh kg^–1^ in Figure 21B. Besides, Yao's group reported several aqueous full batteries with quinone anodes and common inorganic cathodes.^[^ ^86^ ^]^ The pyrene‐4,5,9,10‐tetraone polymer‐LiMn_2_O_4_ cell displayed an average voltage of 1.13 V, yielding an energy density of 92 Wh kg^–1^ and a capacity retention of 80% after 3,000 cycles. Recently, Li et al. reported an optimized quinones/inorganic hybrid battery system in alkali‐acid hybrid electrolyte (Figure 21C).^[^ ^212^ ^]^ The P14AQ/MnO_2_ practical aqueous batteries with an output voltage of 2 V (Figure 21D) showed a capacity of 33 mAh and outstanding electrochemical performance, owing to design for the electrochemical redox couples (fast quinone/phenol coupling with affordable Mn^2+^/MnO_2_ redox reactions) and optimized electrolyte system.

Moreover, organic‐based electrode can also be applied in other energy storage systems (OEMs‐based Li‐S batteries, OEMs‐based zinc air batteries, OEMs‐air batteries, etc.). For OEMs‐based Li‐S batteries, OEMs could be used as the S‐loading hosts, redox mediators, functional separators, etc. For instance, Cui and co‐workers designed a Li_2_S@AQT cell with all‐solid‐state electrolyte, and Li_2_S cathodes with AQT redox mediator presented a reduced energy barrier for activation and superior cycling stability.^[^ ^213^ ^]^ AQT redox mediator promoted the charge transfer rate at the electrode‐electrolyte interface, thus activating the redox reaction of insulting S/Li_2_S speciates. Another example is 2D polyimide COFs used as the S‐loading host for Li‐S batteries by Li's work, and the ultrathin COF nanosheets with redox‐active carbonyls could not only combine polysulfides species through sulfiphilic/lithiophilic interactions but also physically confine polysulfides within well‐defined pores.^[^ ^214^ ^]^ S electrode with polyimide COFs host exhibited excellent cycling stability and rate performance, paving the way for designing novel OEMs with functional groups for high‐performance Li‐S batteries.

In terms of OEMs‐based air batteries, OEMs are mainly applied in the cathode to catalyze the oxygen reduction/evolution reactions. Xiang's research group reported a soluble COF with atomically positive charged centers for Zn‐air battery via in situ charge exfoliation approach.^[^ ^215^ ^]^ The soluble COF contained with N‐coordinated Fe center and closed π‐conjugated structure shows superior catalytic performance for oxygen reduction reaction, providing a soluble COF in a wide range of versatile applications. In addition, benefiting from dendrite‐free and fast redox reaction features, OEMs are likely to be used as anode for the air batteries. Li et al. reported an aqueous polyanthraquinone‐air battery, which exhibited good cycling stability (92% capacity retention after 500 cycles) and superior rate capability (147 mAh g^–1^_P14AQ_, 10 A g^–1^).^[^ ^216^ ^]^ Therefore, the OEMs with structural tunability and functional diversity are potentially universal electrode materials for any secondary battery systems with ecological energy characteristics.

### 4.3 Quasi‐practical batteries

Rechargeable organic batteries with high active material mass loading and limited electrolyte usage are likely an important step toward practical batteries. Mass loading of active material has a great influence on the mass/power energy density and electrochemical performance of quasi‐practical batteries.^[^ ^17^ ^]^ However, the areal capacity of most OEMs is below 1 mAh cm^–2^ in the lab‐level cell. Some efforts have been made to improve the areal capacity of OEMs. For example, Gutel et al. tried to construct an 3,4,9,10‐perylene‐tetracarboxylic‐dianhydride (PTCLi_4_) electrode with 0.5 wt% carbon additive, the PTCLi_4_ electrode with high mass loading (12 mg cm^–2^) showed a high areal capacity of ∼1.2 mAh cm^–2^ and good cycling stability.^[^ ^217^ ^]^ Vlad's research group reported a conjugated sulfonamide (Li_4_‐PTtSA) with a redox potential of 3.45 V, and the Li_4_‐PTtSA‐Li_4_Ti_5_O_12_ full cells (with a Li_4_‐PTtSA mass‐loading of 7 mg cm^–2^) displayed stable cycling over 250 cycles.^[^ ^55^ ^]^ Notably, conjugated sulfonamide materials showed gravimetric energy storage metrics comparable to those of the commercial inorganic electrodes. However, the amounts of the electrolyte usage are not stressed in most of the reported OEMs‐based quasi‐practical batteries, and how to ensure the high energy density of quasi‐practical batteries with limited electrolyte is another point. Apart from the assembly process and cost factors, the next‐generation LIBs with the energy density goal of 500 Wh kg^–1^ require the areal capacity of active material near to 6 mAh cm^–2^ with minimum amounts of electrolyte,^[^ ^218^ ^]^ many efforts on electrode design should be made to promote the lab‐level OEMs‐based batteries to the commercial level.

### 4.4 Biodegradable batteries

**FIGURE 22:**
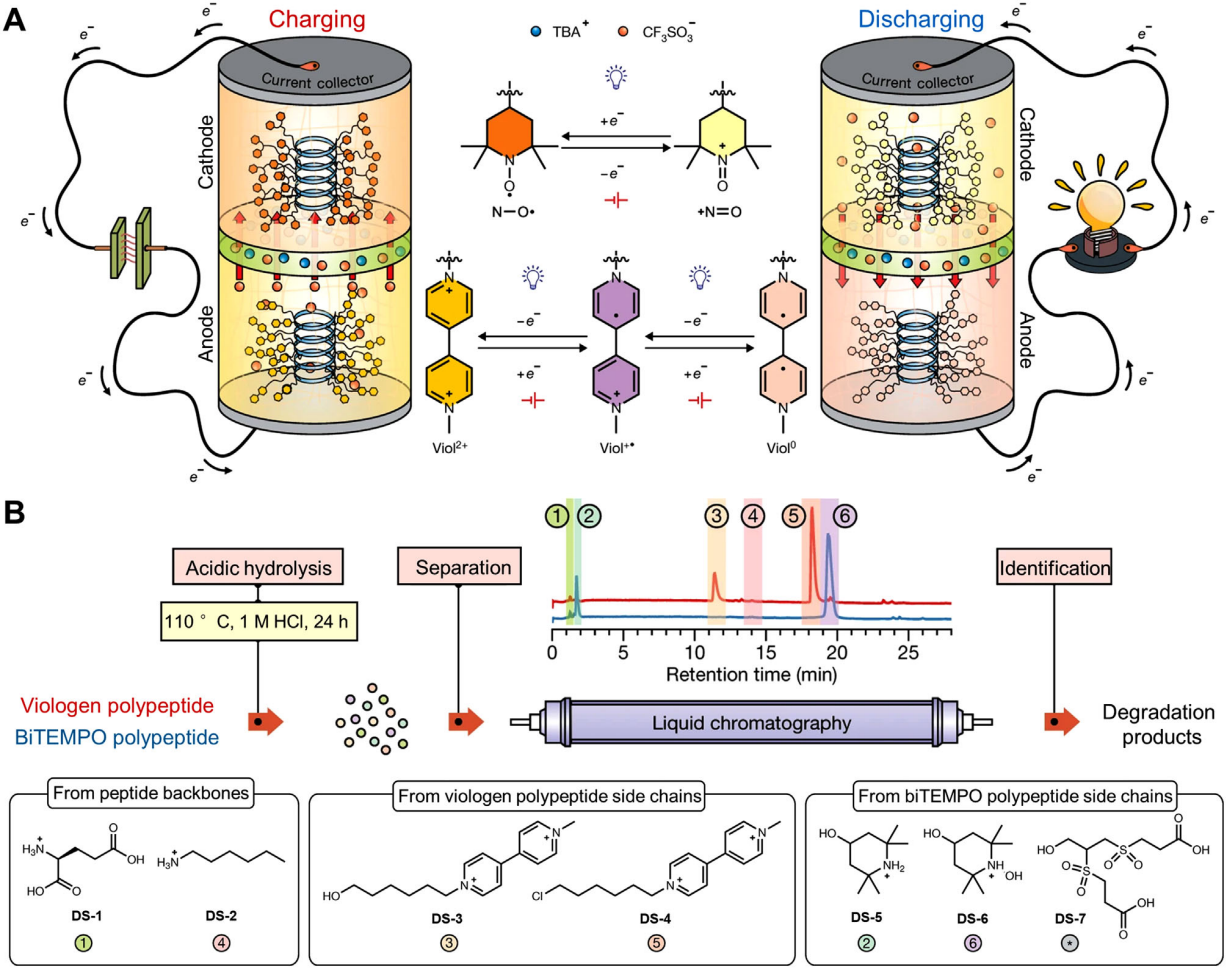
(A) Schematics and redox reactions of the polypeptide‐based organic radical battery. (B) The workflow and structural identification for the complete degradation products of viologen and biTEMPO polypeptides. Reproduced with permission.^[^ ^52^ ^]^ Copyright 2021, Nature Publishing Group

With the rapid expanding of rechargeable batteries, a new challenge is emerging as the recycling and disposal process of electrode materials in an eco‐friendly way. With the increasing strategic demand for global supply material, the environmental and resource challenges for the mineral resources are highlighted.^[^ ^219^ ^]^ Redox‐active OEMs are one type of potential candidates to construct the eco‐friendly and sustainable batteries, owing to their natural origination from biodegradable resources. Some naturally occurring and biodegradable OEMs are employed as electrode materials in rechargeable batteries, but without further separation and recycled of degradation products.^[^ ^43^, ^220^ ^]^ Recently, Wooley and collaborators reported fully polypeptide‐based biodegradable cells with a capacity retention of 85% after 250 cycles, and the viologens and nitroxide radicals along polypeptide backbones were used as the anode and cathode materials (Figure 22A), respectively.^[^ ^52^ ^]^ The identification and separation for degradation species of the viologen and biTEMPO polypeptides were carried out with non‐hazardous disposal (Figure 22B), and the reconstruction methods from the degradation products of polypeptides were proposed in principle. This all‐organic polypeptide‐based battery demonstrates the potential of sustainable and recyclable OEMs‐based rechargeable batteries. Moreover, Moore et al. systematically studied the oxidation‐triggered mesolytic cleavage of redox‐active homobenzylic ethers for the electrochemical degradation of organic flow cell.^[^ ^221^ ^]^ The triggerable retrograde reactions of homobenzylic ethers were applied to break up damaged redoxmer materials, inhibiting the irreparable chemical damage to lifetime and capacity of energy storage devices. The redox‐triggered mesolytic cleavage strategy is promisingly contributed to the programmable end‐of‐life function of developing sustainable OEMs. The biodegradable batteries with recycled and sustainable properties have broad development prospects in energy storage systems, and more efforts should be made to design the eco‐friendly and degradable batteries through structural engineering and machine learning strategies at multiscale levels.

## 5 SUMMARY AND OUTLOOK

OEMs with bountiful resources, structural designability, and sustainability offer an attractive solution to develop the degradable and eco‐friendly batteries, and meet the requirements of low carbon footprint, less energy consumption, and sustainability for next‐generation energy‐storage systems.^[^ ^222^, ^223^ ^]^ Nevertheless, the current redox‐active OEMs still suffer from the issues of inferior electronic conductivity, solubility, lower redox potential, complex redox reaction mechanism, and limited characterization techniques.^[^ ^224^ ^]^ Unlike inorganic electrodes, OEMs with flexible structure and free heavy metal atoms are difficult to be monitored structural transformations through the conventional tools.^[^ ^225^, ^226^ ^]^ Thanks to the functional groups (i.e., C═O, C═N, N═N, S─S) of organic compounds, the in situ spectroscopy measurements (FTIR, UV, Raman, EPR, etc.), combined with novel emerging theoretical calculation methods, are powerful measurements in providing useful information of the structural changes of OEMs.

**FIGURE 23:**
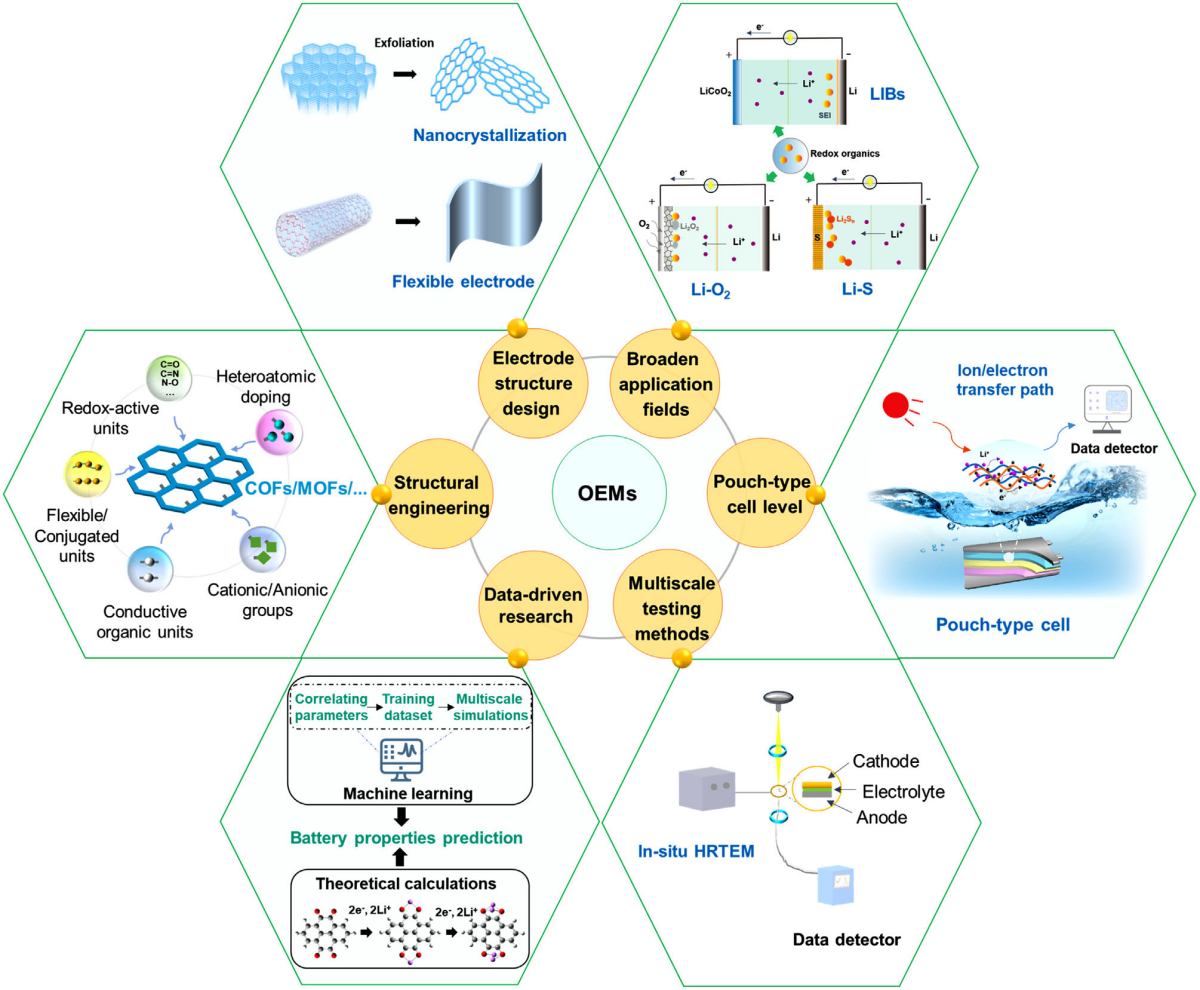
Diagram of advanced strategies to current issues of OEMs for sustainable secondary batteries

To overcome the barriers from fundamental understanding to further commercial applications of organic batteries, great efforts have been done for the comprehensive enhancement on rechargeable organic batteries.^[^ ^227^, ^228^, ^229^ ^]^ Advanced strategies to resolve practical issues of OEMs are summarized in this review, providing possible resolving approaches and suggestions to further understand the redox reaction mechanisms at multiscale levels. As an outlook, the sustainable and flexible organic batteries can be further optimized through the following five aspects (Figure 23):

The current OEMs still face low practical mass energy density and unsatisfactory electrochemical performance, which limit their further applications in future large‐scale energy storage devices. In general, the function‐oriented structural engineering for novel OEMs is an effective solution to construct the high‐performance organic batteries. Besides, the theoretical specific capacity of OEMs can be achieved by introducing less amount of inactive ingredients and the redox potentials, and electronic conductivity of redox‐active organics could be optimized by the structural engineering strategy as well. Moreover, large‐scale production and commercial applications of organic materials are mostly affected by their synthesis routes, cost of raw materials, active material‐loading, gravimetric density, and so on, which are crucial parameters for the applications of organic‐based batteries or pouch‐type cells in future energy storage devices.

OEMs with abundant redox‐active groups, eco‐friendly, and structure designability features are one type of promising candidates for other battery systems as well. For instance, OEMs could be used as the sulfur‐loading substrate, electrochemically active binder, and redox reaction mediums for LIBs, Li‐S battery, and lithium oxygen battery. Besides, the potential effects of OEMs in the electrode structure modification, electrolyte optimization and negative electrode production are also foreseeable, owing to the fast ion transfer rate and flexible chain of organic polymers. Notably, benefiting from good tolerance of flexible organic materials to extreme temperature, the application of OEMs in wide temperature range batteries and solid‐state batteries achieves more and more attention of researchers. Thereby, the broaden applications of OEMs in other battery systems may open up avenues toward the development of more commercial energy storage devices.

Although the redox reaction mechanism of OEMs is elucidated at the molecular level, most of the electron or ion transfer routes and the interface structures between the organic electrode and electrolyte in detail are still elusive. In addition, the existing states and stability of organic intermediates during the ion intercalation/extraction process at multiple length scales are needed to be explored further with high resolution characterization techniques and theoretical calculations. The emerging machine learning‐assisted methodology is promising to ensure the compatibility between efficiency and accuracy of theoretical calculations, speeding up the analysis process of structure changes and redox mechanisms of OEMs. A deeper and further understanding of redox reaction mechanism of OEMs would provide more opportunities for the data‐driven research of organic rechargeable batteries.

Explorations in developing advanced characterization tools for elucidating the reaction mechanism of organic batteries remain challenging for researchers. Inspired by the characterization methods for the inorganic electrodes, in situ monitor tools (i.e., XPS, FTIR, Raman, NMR spectra) are introduced into OEMs area to provide crucial information for determining the redox reaction mechanisms. Moreover, artificial intelligence‐based machine learning methods are effective means to speed up the screening of organic electrodes and clarify the redox mechanism of rechargeable batteries. Novel emerging tools like cryo‐electron microscopy and high resolution TEM, in combination with continuous machine learning‐assisted methods, are likely to witness a boom of OEMs in the areas of electronic devices and digital technologies.

Up to now, the electrochemical performance of OEMs‐based rechargeable batteries, even for the pouch‐type cells and OEMs‐based full batteries, are usually tested under the conditions far from practically relevant ones, which could not meet the industrial requirements. The practical cell design factors (large‐scale production, areal mass loading, safety/tolerance tests, the amount of electrolyte, practical power/energy density, etc.) should be taken into consideration to evaluate the electrochemical performance at a pouch‐type cell level. How to carry out the large‐scale production of OEMs with green synthesis route and low‐cost macrofabrication technology will be attractive, as well as the efficient recycling of OEMs at the cell level. Meanwhile, the corresponding high resolution characterizations for electrode structure and redox mechanism of the cell‐level batteries are needed as well. With the combination of updating characterization and computational techniques, it is hoped that these viewpoints would promote the development of OEMs in practical applications in a near future.

## CONFLICT OF INTEREST

The authors declare no conflict of interest.

## References

[1] J. B. Goodenough , Y. Kim , Chem. Mater. 2010, 22, 587.

[2] M. Winter , B. Barnett , K. Xu , Chem. Rev. 2018, 118, 11433. 30500179 10.1021/acs.chemrev.8b00422

[3] J. W. Choi , D. Aurbach , Nat. Rev. Mater. 2016, 1, 16013.

[4] J. M. Tarascon , M. Armand , Nature 2001, 414, 359. 11713543 10.1038/35104644

[5] E. Fan , L. Li , Z. Wang , J. Lin , Y. Huang , Y. Yao , R. Chen , F. Wu , Chem. Rev. 2020, 120, 7020. 31990183 10.1021/acs.chemrev.9b00535

[6] N. Yabuuchi , K. Kubota , M. Dahbi , S. Komaba , Chem. Rev. 2014, 114, 11636. 25390643 10.1021/cr500192f

[7] N. Yabuuchi , M. Kajiyama , J. Iwatate , H. Nishikawa , S. Hitomi , R. Okuyama , R. Usui , Y. Yamada , S. Komaba , Nat. Mater. 2012, 11, 512. 22543301 10.1038/nmat3309

[8] J. Liu , J. Wang , Y. Ni , K. Zhang , F. Cheng , J. Chen , Mater. Today 2021, 43, 132.

[9] L. Sharma , S. P. Adiga , H. N. Alshareef , P. Barpanda , Adv. Energy Mater. 2020, 10, 2001449.

[10] J. Qian , C. Wu , Y. Cao , Z. Ma , Y. Huang , X. Ai , H. Yang , Adv. Energy Mater. 2018, 8, 1702619.

[11] K. Turcheniuk , D. Bondarev , V. Singhal , G. Yushin , Nature 2018, 559, 467. 30046087 10.1038/d41586-018-05752-3

[12] T. Liu , J. P. Vivek , E. W. Zhao , J. Lei , N. Garcia‐Araez , C. P. Grey , Chem. Rev. 2020, 120, 6558. 32090540 10.1021/acs.chemrev.9b00545

[13] Z. W. Seh , Y. Sun , Q. Zhang , Y. Cui , Chem. Soc. Rev. 2016, 45, 5605. 27460222 10.1039/c5cs00410a

[14] T. B. Schon , B. T. McAllister , P.‐F. Li , D. S. Seferos , Chem. Soc. Rev. 2016, 45, 6345. 27273252 10.1039/c6cs00173d

[15] D. Luo , M. Li , Q. Ma , G. Wen , H. Dou , B. Ren , Y. Liu , X. Wang , L. Shui , Z. Chen , Chem. Soc. Rev. 2022, 51, 2917. 35285470 10.1039/d1cs01014j

[16] Y. Li , W. Chen , G. Xing , D. Jiang , L. Chen , Chem. Soc. Rev. 2020, 49, 2852. 32377651 10.1039/d0cs00199f

[17] Y. Lu , J. Chen , Nat. Rev. Chem. 2020, 4, 127. 37128020 10.1038/s41570-020-0160-9

[18] Z. Song , H. Zhou , Energy Environ. Sci. 2013, 6, 2280.

[19] D.‐G. Wang , T. Qiu , W. Guo , Z. Liang , H. Tabassum , D. Xia , R. Zou , Energy Environ. Sci. 2021, 14, 688.

[20] D. L. Williams , J. J. Byrne , J. S. Driscoll , J. Electrochem. Soc. 1969, 116, 2.

[21] H. Alt , H. Binder , A. Köhling , G. Sandstede , Electrochim. Acta 1972, 17, 873.

[22] H. Shirakawa , E. J. Louis , A. G. Macdiarmid , C. K. Chiang , A. J. Heeger , J. Chem. Soc., Chem. Commun. 1977, 16, 578.

[23] X. Guo , A. Facchetti , Nat. Mater. 2020, 19, 922. 32820293 10.1038/s41563-020-0778-5

[24] D. MacInnes , M. A. Druy , P. J. Nigrey , D. P. Nairns , A. G. MacDiarmid , A. J. Heeger , J. Chem. Soc., Chem. Commun. 1981, 7, 317.

[25] H. Gao , L. Xue , S. Xin , J. B. Goodenough , Angew. Chem. Int. Ed. 2018, 57, 5449. 10.1002/anie.201802248 29534324

[26] S. Lee , G. Kwon , K. Ku , K. Yoon , S.‐K. Jung , H.‐D. Lim , K. Kang , Adv. Mater. 2018, 30, 1704682. 10.1002/adma.201704682 29582467

[27] I. Aldalur , M. Armand , H. Zhang , Batteries Supercaps 2020, 3, 30.

[28] H. Li , M. Tang , Y. Wu , Y. Chen , S. Zhu , B. Wang , C. Jiang , E. Wang , C. Wang , J. Phys. Chem. Lett. 2018, 9, 3205. 29846068 10.1021/acs.jpclett.8b01285

[29] H. Dong , Y. Liang , O. Tutusaus , R. Mohtadi , Y. Zhang , F. Hao , Y. Yao , Joule 2019, 3, 782.

[30] F. Xu , S. Jin , H. Zhong , D. Wu , X. Yang , X. Chen , H. Wei , R. Fu , D. Jiang , Sci. Rep. 2015, 5, 8225. 25650133 10.1038/srep08225 PMC4316169

[31] R. Liu , K. T. Tan , Y. Gong , Y. Chen , Z. Li , S. Xie , T. He , Z. Lu , H. Yang , D. Jiang , Chem. Soc. Rev. 2021, 50, 120. 33283811 10.1039/d0cs00620c

[32] K. Qin , J. Huang , K. Holguin , C. Luo , Energy Environ. Sci. 2020, 13, 3950.

[33] K. Nakahara , S. Iwasa , M. Satoh , Y. Morioka , J. Iriyama , M. Suguro , E. Hasegawa , Chem. Phys. Lett. 2002, 359, 351.

[34] R. Rajagopalan , Y. Tang , C. Jia , X. Ji , H. Wang , Energy Environ. Sci. 2020, 13, 1568.

[35] H. Zhao , W. A. Lam , L. Wang , H. Xu , W. A. Daoud , X. He , Energy Environ. Sci. 2022, 15, 2329.

[36] B. Esser , F. Dolhem , M. Becuwe , P. Poizot , A. Vlad , D. Brandell , J. Power Sources 2021, 482, 228814.

[37] Y. Lu , L. Li , Q. Zhang , Z. Niu , J. Chen , Joule 2018, 2, 1747.

[38] M. E. Bhosale , S. Chae , J. M. Kim , J.‐Y. Choi , J. Mater. Chem. A 2018, 6, 19885.

[39] Y. Chen , C. Wang , Acc. Chem. Res. 2020, 53, 2636. 32976710 10.1021/acs.accounts.0c00465

[40] P. Yu , Y. Zeng , H. Zhang , M. Yu , Y. Tong , X. Lu , Small 2019, 15, 1804760. 10.1002/smll.201804760 30667603

[41] X. Fan , F. Wang , X. Ji , R. Wang , T. Gao , S. Hou , J. Chen , T. Deng , X. Li , L. Chen , C. Luo , L. Wang , C. Wang , Angew. Chem. Int. Ed. 2018, 57, 7146. 10.1002/anie.201803703 29704298

[42] T. Matsunaga , T. Kubota , T. Sugimoto , M. Satoh , Chem. Lett. 2011, 40, 750.

[43] Q. Zhao , W. Huang , Z. Luo , L. Liu , Y. Lu , Y. Li , L. Li , J. Hu , H. Ma , J. Chen , Sci. Adv. 2018, 4, eaao1761. 29511734 10.1126/sciadv.aao1761 PMC5837429

[44] Y. Li , L. Liu , Y. Lu , R. Shi , Y. Ma , Z. Yan , K. Zhang , J. Chen , Adv. Funct. Mater. 2021, 31, 2102063.

[45] S. Cao , H. Zhang , Y. Zhao , Y. Zhao , eScience 2021, 1, 28.

[46] B. M. Peterson , C. N. Gannett , L. Melecio‐Zambrano , B. P. Fors , H. Abruña , ACS Appl. Mater. Interfaces 2021, 13, 7135. 33543926 10.1021/acsami.0c19622

[47] Y. Liu , Z. Niu , G. Dai , Y. Chen , H. Li , L. Huang , X. Zhang , Y. Xu , Y. Zhao , Mater. Today Energy 2021, 21, 100812.

[48] H. Kye , Y. Kang , D. Jang , J. E. Kwon , B.‐G. Kim , Adv. Energy Sustainability Res. 2022, 2200030. 10.1002/aesr.202200030

[49] Y. Xie , K. Zhang , Y. Yamauchi , K. Oyaizu , Z. Jia , Mater. Horiz. 2021, 8, 803. 34821316 10.1039/d0mh01391a

[50] S. Xu , H. Dai , S. Zhu , Y. Wu , M. Sun , Y. Chen , K. Fan , C. Zhang , C. Wang , W. Hu , eScience 2021, 1, 60.

[51] H. Chen , C. Xu , Y. Zhang , M. Cao , H. Dou , X. Zhang , Batteries Supercaps 2019, 2, 979.

[52] T. P. Nguyen , A. D. Easley , N. Kang , S. Khan , S.‐M. Lim , Y. H. Rezenom , S. Wang , D. K. Tran , J. Fan , R. A. Letteri , X. He , L. Su , C.‐H. Yu , J. L. Lutkenhaus , K. L. Wooley , Nature 2021, 593, 61. 33953410 10.1038/s41586-021-03399-1

[53] Y. Zhao , M. Wu , H. Zhang , Z. Ge , C. Li , Y. Ma , Y. Chen , Energy Storage Mater. 2022, 47, 141.

[54] Y. Liang , Y. Yao , Joule 2018, 2, 1690.

[55] J. Wang , A. E. Lakraychi , X. Liu , L. Sieuw , C. Morari , P. Poizot , A. Vlad , Nat. Mater. 2021, 20, 665. 33318677 10.1038/s41563-020-00869-1

[56] M. Wu , Y. Zhao , B. Sun , Z. Sun , C. Li , Y. Han , L. Xu , Z. Ge , Y. Ren , M. Zhang , Q. Zhang , Y. Lu , W. Wang , Y. Ma , Y. Chen , Nano Energy 2020, 70, 104498.

[57] Z. Tian , V. S. Kale , Y. Wang , S. Kandambeth , J. Czaban‐Jóźwiak , O. Shekhah , M. Eddaoudi , H. N. Alshareef , J. Am. Chem. Soc. 2021, 143, 19178. 34739750 10.1021/jacs.1c09290

[58] W. Wang , V. S. Kale , Z. Cao , Y. Lei , S. Kandambeth , G. Zou , Y. Zhu , E. Abouhamad , O. Shekhah , L. Cavallo , M. Eddaoudi , H. N. Alshareef , Adv. Mater. 2021, 33, 2103617. 10.1002/adma.202103617 34365688

[59] L. Wang , T. Liu , T. Wu , J. Lu , Exploration 2021, 1, 20210130. 10.1002/EXP.20210130 PMC10190967 37323695

[60] C. P. Grey , D. S. Hall , Nat. Commun. 2020, 11, 6279. 33293543 10.1038/s41467-020-19991-4 PMC7722877

[61] G. Harper , R. Sommerville , E. Kendrick , L. Driscoll , P. Slater , R. Stolkin , A. Walton , P. Christensen , O. Heidrich , S. Lambert , A. Abbott , K. Ryder , L. Gaines , P. Anderson , Nature 2019, 575, 75. 31695206 10.1038/s41586-019-1682-5

[62] F. Wu , J. Maier , Y. Yu , Chem. Soc. Rev. 2020, 49, 1569. 32055806 10.1039/c7cs00863e

[63] J. Xie , P. Gu , Q. Zhang , ACS Energy Lett. 2017, 2, 1985.

[64] Y. Liang , P. Zhang , S. Yang , Z. Tao , J. Chen , Adv. Energy Mater. 2013, 3, 600.

[65] S. Xu , J. Liang , Y. Yu , R. Liu , Y. Xu , X. Zhu , Y. Zhao , J. Phys. Chem. C 2021, 125, 21352.

[66] S. Muench , A. Wild , C. Friebe , B. Häupler , T. Janoschka , U. S. Schubert , Chem. Rev. 2016, 116, 9438. 27479607 10.1021/acs.chemrev.6b00070

[67] T. Kim , W. Song , D.‐Y. Son , L. K. Ono , Y. Qi , J. Mater. Chem. A 2019, 7, 2942.

[68] Y. Zhang , L. Tao , C. Xie , D. Wang , Y. Zou , R. Chen , Y. Wang , C. Jia , S. Wang , Adv. Mater. 2020, 32, 1905923. 10.1002/adma.201905923 31930593

[69] L. Li , Y. Yin , J. Hei , X. Wan , M. Li , Y. Cui , Small 2021, 17, 2005752. 10.1002/smll.202005752 33544971

[70] Q. Zhao , Y. Lu , J. Chen , Adv. Energy Mater. 2017, 7, 1601792.

[71] Y. Xu , M. Zhou , Y. Lei , Mater. Today 2018, 21, 60.

[72] Y. Lu , Y. Cai , Q. Zhang , J. Chen , Adv. Mater. 2021, 34, 2104150. 10.1002/adma.202104150 34617334

[73] Y. Lu , X. Hou , L. Miao , L. Li , R. Shi , L. Liu , J. Chen , Angew. Chem. Int. Ed. 2019, 58, 7020. 10.1002/anie.201902185 30916877

[74] C. Yao , Z. Wu , J. Xie , F. Yu , W. Guo , Z. J. Xu , D. Li , S. Zhang , Q. Zhang , ChemSusChem 2020, 13, 2457. 31782976 10.1002/cssc.201903007

[75] C. Peng , G.‐H. Ning , J. Su , G. Zhong , W. Tang , B. Tian , C. Su , D. Yu , L. Zu , J. Yang , M.‐F. Ng , Y.‐S. Hu , Y. Yang , M. Armand , K. P. Loh , Nat. Energy 2017, 2, 17074.

[76] R. P. Fornari , P. Silva , Wiley Interdiscip. Rev. Comput. Mol. Sci. 2021, 11, e1495.

[77] W. Li , B. Song , A. Manthiram , Chem. Soc. Rev. 2017, 46, 3006. 28440379 10.1039/c6cs00875e

[78] S. Lee , K. Lee , K. Ku , J. Hong , S. Y. Park , J. E. Kwon , K. Kang , Adv. Energy Mater. 2020, 10, 2001635.

[79] D. Tomerini , C. Gatti , C. Frayret , Phys. Chem. Chem. Phys. 2015, 17, 8604. 25738747 10.1039/c4cp05998k

[80] K. Lee , I. E. Serdiuk , G. Kwon , D. J. Min , K. Kang , S. Y. Park , J. E. Kwon , Energy Environ. Sci. 2020, 13, 4142.

[81] S. Wang , Q. Wang , P. Shao , Y. Han , X. Gao , L. Ma , S. Yuan , X. Ma , J. Zhou , X. Feng , B. Wang , J. Am. Chem. Soc. 2017, 139, 4258. 28316238 10.1021/jacs.7b02648

[82] D. Shen , A. M. Rao , J. Zhou , B. Lu , Angew. Chem. Int. Ed. 2022, 61, e202201972. 10.1002/anie.202201972 35294100

[83] J. Huang , X. Dong , Z. Guo , Y. Wang , Angew. Chem. Int. Ed. 2020, 59, 18322. 10.1002/anie.202003198 32329546

[84] Z. Tie , Z. Niu , Angew. Chem. Int. Ed. 2020, 59, 21293. 10.1002/anie.202008960 32692428

[85] Z. Liu , Y. Huang , Y. Huang , Q. Yang , X. Li , Z. Huang , C. Zhi , Chem. Soc. Rev. 2020, 49, 180. 31781706 10.1039/c9cs00131j

[86] Y. Liang , Y. Jing , S. Gheytani , K.‐Y. Lee , P. Liu , A. Facchetti , Y. Yao , Nat. Mater. 2017, 16, 841. 28628121 10.1038/nmat4919

[87] Y. Luo , F. Zheng , L. Liu , K. Lei , X. Hou , G. Xu , H. Meng , J. Shi , F. Li , ChemSusChem 2020, 13, 2239. 32022410 10.1002/cssc.201903083

[88] Z. Guo , Y. Ma , X. Dong , J. Huang , Y. Wang , Y. Xia , Angew. Chem. Int. Ed. 2018, 53, 11737. 10.1002/anie.201807121 30019809

[89] J. Chen , Q. Zhu , L. Jiang , R. Liu , Y. Yang , M. Tang , J. Wang , H. Wang , L. Guo , Angew. Chem. Int. Ed. 2021, 60, 5794. 10.1002/anie.202011144 33314518

[90] R. Emanuelsson , M. Sterby , M. Strømme , M. Sjödin , J. Am. Chem. Soc. 2017, 139, 4828. 28293954 10.1021/jacs.7b00159

[91] K. Zhang , C. Guo , Q. Zhao , Z. Niu , J. Chen , Adv. Sci. 2015, 2, 1500018. 10.1002/advs.201500018 PMC5115363 27980937

[92] Y. Xie , K. Zhang , Y. Yamauchi , Z. Jia , Polym. Chem. 2020, 11, 4155.

[93] Q. Yu , Z. Xue , M. Li , P. Qiu , C. Li , S. Wang , J. Yu , H. Nara , J. Na , Y. Yamauchi , Adv. Energy Mater. 2021, 11, 2002523.

[94] Q. Zhao , Z. Zhu , J. Chen , Adv. Mater. 2017, 29, 1607007. 10.1002/adma.201607007 28370809

[95] M. Tang , C. Jiang , S. Liu , X. Li , Y. Chen , Y. Wu , J. Ma , C. Wang , Energy Storage Mater. 2020, 27, 35.

[96] T. Ma , L. Liu , J. Wang , Y. Lu , J. Chen , Angew. Chem. Int. Ed. 2020, 59, 11533. 10.1002/anie.202002773 32297392

[97] H. Chen , M. Armand , G. Demailly , F. Dolhem , P. Poizot , J.‐M. Tarascon , ChemSusChem 2008, 1, 348. 18605101 10.1002/cssc.200700161

[98] S. Wang , L. Wang , Z. Zhu , Z. Hu , Q. Zhao , J. Chen , Angew. Chem. Int. Ed. 2014, 53, 5892. 10.1002/anie.201400032 24677513

[99] Y. Wang , P. Bai , B. Li , C. Zhao , Z. Chen , M. Li , H. Su , J. Yang , Y. Xu , Adv. Energy Mater. 2021, 11, 2101972.

[100] S. Bai , B. Kim , C. Kim , O. Tamwattana , H. Park , J. Kim , D. Lee , K. Kang , Nat. Nanotechnol. 2021, 16, 77. 33139935 10.1038/s41565-020-00788-x

[101] M. Li , J. Yang , Y. Shi , Z. Chen , P. Bai , H. Su , P. Xiong , M. Cheng , J. Zhao , Y. Xu , Adv. Mater. 2021, 34, 2107226. 10.1002/adma.202107226 34796556

[102] M. Lee , J. Hong , J. Lopez , Y. Sun , D. Feng , K. Lim , W. C. Chueh , M. F. Toney , Y. Cui , Z. Bao , Nat. Energy 2017, 2, 861.

[103] Y. Liang , H. Dong , D. Aurbach , Y. Yao , Nat. Energy 2020, 5, 646.

[104] C. Luo , X. Ji , J. Chen , K. J. Gaskell , X. He , Y. Liang , J. Jiang , C. Wang , Angew. Chem. Int. Ed. 2018, 57, 8567. 10.1002/anie.201804068 29791780

[105] J. Wan , J. Xie , D. G. Mackanic , W. Burke , Z. Bao , Y. Cui , Mater. Today Nano 2018, 4, 1.

[106] F. Wang , E. Hu , W. Sun , T. Gao , X. Ji , X. Fan , F. Han , X.‐Q. Yang , K. Xu , C. Wang , Energy Environ. Sci. 2018, 11, 3168.

[107] H. Chen , Z. Zhang , Z. Wei , G. Chen , X. Yang , C. Wang , F. Du , Sustainable Energy Fuels 2020, 4, 128.

[108] X. Wang , Z. Shang , A. Yang , Q. Zhang , F. Cheng , D. Jia , J. Chen , Chem 2019, 5, 364.

[109] F. Hao , X. Chi , Y. Liang , Y. Zhang , R. Xu , H. Guo , T. Terlier , H. Dong , K. Zhao , J. Lou , Y. Yao , Joule 2019, 3, 1349.

[110] H. Chen , M. Zheng , S. Qian , H. Y. Ling , Z. Wu , X. Liu , C. Yan , S. Zhang , Carbon Energy 2021, 3, 929.

[111] M. Wu , Y. Cui , A. Bhargav , Y. Losovyj , A. Siegel , M. Agarwal , Y. Ma , Y. Fu , Angew. Chem. Int. Ed. 2016, 55, 10027. 10.1002/anie.201603897 27411083

[112] A. Molina , N. Patil , E. Ventosa , M. Liras , J. Palma , R. Marcilla , ACS Energy Lett. 2020, 5, 2945.

[113] R. R. Kapaev , I. S. Zhidkov , E. Z. Kurmaev , K. J. Stevenson , P. A. Troshin , J. Mater. Chem. A 2019, 7, 22596.

[114] H.‐N. Wang , X. Meng , L.‐Z. Dong , Y. Chen , S.‐L. Li , Y.‐Q. Lan , J. Mater. Chem. A 2019, 7, 24059.

[115] F. Zhao , Y. Shi , L. Pan , G. Yu , Acc. Chem. Res. 2017, 50, 1734. 28649845 10.1021/acs.accounts.7b00191

[116] Z. Song , Y. Qian , T. Zhang , M. Otani , H. Zhou , Adv. Sci. 2015, 2, 1500124. 10.1002/advs.201500124 PMC5115381 27980977

[117] X. Dong , L. Chen , J. Liu , S. Haller , Y. Wang , Y. Xia , Sci. Adv. 2016, 2, e1501038. 26844298 10.1126/sciadv.1501038 PMC4737207

[118] Z. Song , Y. Qian , X. Liu , T. Zhang , Y. Zhu , H. Yu , M. Otani , H. Zhou , Energy Environ. Sci. 2014, 7, 4077.

[119] Z. Song , Y. Qian , M. L. Gordin , D. Tang , T. Xu , M. Otani , H. Zhan , H. Zhou , D. Wang , Angew. Chem. Int. Ed. 2015, 54, 13947. 10.1002/anie.201506673 26411505

[120] M. Tang , S. Zhu , Z. Liu , C. Jiang , Y. Wu , H. Li , B. Wang , E. Wang , J. Ma , C. Wang , Chem 2018, 4, 2600.

[121] Z. Wang , W. Jin , X. Huang , G. Lu , Y. Li , Chem. Rec. 2020, 20, 1198. 32881320 10.1002/tcr.202000074

[122] J. Li , X. Jing , Q. Li , S. Li , X. Gao , X. Feng , B. Wang , Chem. Soc. Rev. 2020, 49, 3565. 32369058 10.1039/d0cs00017e

[123] Y. Liu , W. Zhou , W. L. Teo , K. Wang , L. Zhang , Y. Zeng , Y. Zhao , Chem 2020, 6, 3172.

[124] R. Shi , L. Liu , Y. Lu , C. Wang , Y. Li , L. Li , Z. Yan , J. Chen , Nat. Commun. 2020, 11, 178. 31924753 10.1038/s41467-019-13739-5 PMC6954217

[125] M. Mao , C. Luo , T. P. Pollard , S. Hou , T. Gao , X. Fan , C. Cui , J. Yue , Y. Tong , G. Yang , T. Deng , M. Zhang , J. Ma , L. Suo , O. Borodin , C. Wang , Angew. Chem. Int. Ed. 2019, 58, 17820. 10.1002/anie.201910916 31571354

[126] M. Li , J. Liu , Y. Li , G. Xing , X. Yu , C. Peng , L. Chen , CCS Chem. 2021, 3, 696.

[127] S. Cheng , W. Gao , Z. Cao , Y. Yang , E. Xie , J. Fu , Adv. Mater. 2022, 34, 2109870. 10.1002/adma.202109870 35112396

[128] S. Umezawa , T. Douura , K. Yoshikawa , D. Tanaka , V. Stolojan , S. R. P. Silva , M. Yoneda , K. Gotoh , Y. Hayashi , Energy Environ. Mater. 2022, *0*, 1‐13. 10.1002/EEM2.12320

[129] Q. Jiang , P. Xiong , J. Liu , Z. Xie , Q. Wang , X. Yang , E. Hu , Y. Cao , J. Sun , Y. Xu , L. Chen , Angew. Chem. Int. Ed. 2020, 59, 5273. 10.1002/anie.201914395 31893570

[130] Y. Chen , Q. Zhu , K. Fan , Y. Gu , M. Sun , Z. Li , C. Zhang , Y. Wu , Q. Wang , S. Xu , J. Ma , C. Wang , W. Hu , Angew. Chem. Int. Ed. 2021, 60, 18769. 10.1002/anie.202106055 34137139

[131] J. Yan , Y. Cui , M. Xie , G. Yang , D. Bin , D. Li , Angew. Chem. Int. Ed. 2021, 60, 24467. 10.1002/anie.202110373 34519413

[132] S. H. Je , H. J. Kim , J. Kim , J. W. Choi , A. Coskun , Adv. Funct. Mater. 2017, 27, 1703947.

[133] X. Wang , Y. Yang , C. Lai , R. Li , H. Xu , D. H. S. Tan , K. Zhang , W. Yu , O. Fjeldberg , M. Lin , W. Tang , Y. S. Meng , K. P. Loh , Angew. Chem. Int. Ed. 2021, 60, 11359. 10.1002/anie.202016240 33751750

[134] L. Zhou , D. L. Danilov , R. Eichel , P. H. L. Notten , Adv. Energy Mater. 2021, 11, 2001304.

[135] C. Niu , W. Luo , C. Dai , C. Yu , Y. Xu , Angew. Chem. Int. Ed. 2021, 60, 24915. 10.1002/anie.202107444 34296502

[136] D. Guo , D. B. Shinde , W. Shin , E. Abou‐Hamad , A. Emwas , Z. Lai , A. Manthiram , Adv. Mater. 2022, 34, 2201410. 10.1002/adma.202201410 35332970

[137] Z. Chang , H. Yang , X. Zhu , P. He , H. Zhou , Nat. Commun. 2022, 13, 1510. 35314688 10.1038/s41467-022-29118-6 PMC8938510

[138] W.‐J. Ong , L.‐L. Tan , Y. H. Ng , S.‐T. Yong , S.‐P. Chai , Chem. Rev. 2016, 116, 7159. 27199146 10.1021/acs.chemrev.6b00075

[139] X. Chen , Y. Li , L. Wang , Y. Xu , A. Nie , Q. Li , F. Wu , W. Sun , X. Zhang , R. Vajtai , P. M. Ajayan , L. Chen , Y. Wang , Adv. Mater. 2019, 31, 1901640. 10.1002/adma.201901640 31155765

[140] Y. Liu , Y. Zhu , Y. Cui , Nat. Energy 2019, 4, 540.

[141] Z. Zhao‐Karger , P. Gao , T. Ebert , S. Klyatskaya , Z. Chen , M. Ruben , M. Fichtner , Adv. Mater. 2019, 31, 1806599. 10.1002/adma.201806599 30786067

[142] P. Jiménez , E. Levillain , O. Alévêque , D. Guyomard , B. Lestriez , J. Gaubicher , Angew. Chem. Int. Ed. 2017, 56, 1553. 10.1002/anie.201607820 28044392

[143] P. Hu , X. He , M. Ng , J. Ye , C. Zhao , S. Wang , K. Tan , A. Chaturvedi , H. Jiang , C. Kloc , W. Hu , Y. Long , Angew. Chem. Int. Ed. 2019, 58, 13513. 10.1002/anie.201906301 31317598

[144] F. Li , Y. Si , Z. Li , W. Guo , Y. Fu , J. Mater. Chem. A 2020, 8, 87.

[145] Y. Li , S. Zheng , X. Liu , P. Li , L. Sun , R. Yang , S. Wang , Z. Wu , X. Bao , W. Deng , Angew. Chem. Int. Ed. 2018, 57, 7992. 10.1002/anie.201711169 29135063

[146] J. L. Segura , R. Juárez , M. Ramos , C. Seoane , Chem. Soc. Rev. 2015, 44, 6850. 26168289 10.1039/c5cs00181a

[147] J. Mahmood , E. K. Lee , M. Jung , D. Shin , I.‐Y. Jeon , S.‐M. Jung , H.‐J. Choi , J.‐M. Seo , S.‐Y. Bae , S.‐D. Sohn , N. Park , J. H. Oh , H.‐J. Shin , J.‐B. Baek , Nat. Commun. 2015, 6, 6486. 25744355 10.1038/ncomms7486 PMC4366516

[148] S. Xu , G. Wang , B. P. Biswal , M. Addicoat , S. Paasch , W. Sheng , X. Zhuang , E. Brunner , T. Heine , R. Berger , X. Feng , Angew. Chem. Int. Ed. 2019, 58, 849. 10.1002/anie.201812685 30461145

[149] A. Jouhara , N. Dupré , A.‐C. Gaillot , D. Guyomard , F. Dolhem , P. Poizot , Nat. Commun. 2018, 9, 4401. 30353001 10.1038/s41467-018-06708-x PMC6199296

[150] J. Wu , X. Rui , G. Long , W. Chen , Q. Yan , Q. Zhang , Angew. Chem. Int. Ed. 2015, 54, 7354. 10.1002/anie.201503072 25960289

[151] S. Wan , J. Guo , J. Kim , H. Ihee , D. Jiang , Angew. Chem. Int. Ed. 2008, 120, 8958.

[152] D. Sheberla , J. C. Bachman , J. S. Elias , C.‐J. Sun , Y. Shao‐Horn , M. Dincă , Nat. Mater. 2017, 16, 220. 27723738 10.1038/nmat4766

[153] P. Xiao , Y. Xu , J. Mater. Chem. A 2018, 6, 21676.

[154] G. Li , B. Zhang , J. Wang , H. Zhao , W. Ma , L. Xu , W. Zhang , K. Zhou , Y. Du , G. He , Angew. Chem. Int. Ed. 2019, 58, 8468. 10.1002/anie.201903152 30951238

[155] J. Park , M. Lee , D. Feng , Z. Huang , A. C. Hinckley , A. Yakovenko , X. Zou , Y. Cui , Z. Bao , J. Am. Chem. Soc. 2018, 140, 10315. 30041519 10.1021/jacs.8b06020

[156] J. Xie , Q. Zhang , Small 2019, 15, 1805061. 10.1002/smll.201805061 30848095

[157] H.‐N. Wang , M. Zhang , A.‐M. Zhang , F.‐C. Shen , X.‐K. Wang , S.‐N. Sun , Y.‐J. Chen , Y.‐Q. Lan , ACS Appl. Mater. Interfaces 2018, 10, 32265. 30175579 10.1021/acsami.8b12194

[158] D. Feng , T. Lei , M. R. Lukatskaya , J. Park , Z. Huang , M. Lee , L. Shaw , S. Chen , A. A. Yakovenko , A. Kulkarni , J. Xiao , K. Fredrickson , J. B. Tok , X. Zou , Y. Cui , Z. Bao , Nat. Energy 2018, 3, 30.

[159] H. Zhang , W. Sun , X. Chen , Y. Wang , ACS Nano 2019, 13, 14252. 31794178 10.1021/acsnano.9b07360

[160] Z. Song , T. Xu , M. L. Gordin , Y.‐B. Jiang , I.‐T. Bae , Q. Xiao , H. Zhan , J. Liu , D. Wang , Nano Lett. 2012, 12, 2205. 22449138 10.1021/nl2039666

[161] E. Vitaku , C. N. Gannett , K. L. Carpenter , L. Shen , H. D. Abruña , W. R. Dichtel , J. Am. Chem. Soc. 2020, 142, 16. 31820958 10.1021/jacs.9b08147

[162] C. Zhao , Z. Chen , W. Wang , P. Xiong , B. Li , M. Li , J. Yang , Y. Xu , Angew. Chem. Int. Ed. 2020, 59, 11992. 10.1002/anie.202000566 32266770

[163] Y. Liu , G. Sun , X. Cai , F. Yang , C. Ma , M. Xue , X. Tao , J. Energy Chem. 2021, 54, 179.

[164] W. Cao , J. Zhang , H. Li , Energy Storage Mater. 2020, 26, 46.

[165] S. Gu , S. Wu , L. Cao , M. Li , N. Qin , J. Zhu , Z. Wang , Y. Li , Z. Li , J. Chen , Z. Lu , J. Am. Chem. Soc. 2019, 141, 9623. 31121094 10.1021/jacs.9b03467

[166] K. Zhang , Y. Xie , M. J. Monteiro , Z. Jia , Energy Storage Mater. 2021, 35, 122.

[167] E. W. Zhao , E. Jónsson , R. B. Jethwa , D. Hey , D. Lyu , A. Brookfield , P. A. A. Klusener , D. Collison , C. P. Grey , J. Am. Chem. Soc. 2021, 143, 1885. 33475344 10.1021/jacs.0c10650 PMC7877726

[168] R. Shi , L. Liu , Y. Lu , Y. Li , S. Zheng , Z. Yan , K. Zhang , J. Chen , Adv. Energy Mater. 2021, 11, 2002917.

[169] M. Lucero , S. Qiu , Z. Feng , Carbon Energy 2021, 3, 762.

[170] P. Poizot , J. Gaubicher , S. Renault , L. Dubois , Y. Liang , Y. Yao , Chem. Rev. 2020, 120, 6490. 32207919 10.1021/acs.chemrev.9b00482

[171] Z. Luo , L. Liu , J. Ning , K. Lei , Y. Lu , F. Li , J. Chen , Angew. Chem. Int. Ed. 2018, 57, 9443. 10.1002/anie.201805540 29863784

[172] M. Zhang , W. Zhou , W. Huang , J. Energy Chem. 2021, 57, 291.

[173] S. Wang , L. Wang , K. Zhang , Z. Zhu , Z. Tao , J. Chen , Nano Lett. 2013, 13, 4404. 23978244 10.1021/nl402239p

[174] Z. Lei , Q. Yang , Y. Xu , S. Guo , W. Sun , H. Liu , L.‐P. Lv , Y. Zhang , Y. Wang , Nat. Commun. 2018, 9, 576. 29422540 10.1038/s41467-018-02889-7 PMC5805684

[175] J. Lu , T. Wu , K. Amine , Nat. Energy 2017, 2, 17011.

[176] W. Yao , W. Zheng , J. Xu , C. Tian , K. Han , W. Sun , S. Xiao , ACS Nano 2021, 15, 7114. 33764730 10.1021/acsnano.1c00270

[177] C. Wang , Y. Fang , Y. Xu , L. Liang , M. Zhou , H. Zhao , Y. Lei , Adv. Funct. Mater. 2016, 26, 1777.

[178] E. Korin , N. Froumin , S. Cohen , ACS Biomater. Sci. Eng. 2017, 3, 882. 33429560 10.1021/acsbiomaterials.7b00040

[179] Y. Li , K. Wang , W. Zhou , Y. Li , R. Vila , W. Huang , H. Wang , G. Chen , G.‐H. Wu , Y. Tsao , H. Wang , R. Sinclair , W. Chiu , Y. Cui , Matter 2019, 1, 428. 34104881 10.1016/j.matt.2019.06.001 PMC8184120

[180] T. Sun , J. Xie , W. Guo , D. Li , Q. Zhang , Adv. Energy Mater. 2020, 10, 1904199.

[181] A. Manthiram , ACS Cent. Sci. 2017, 3, 1063. 29104922 10.1021/acscentsci.7b00288 PMC5658750

[182] Y. Liang , Z. Tao , J. Chen , Adv. Energy Mater. 2012, 2, 742.

[183] Z. Wang , Z. Sun , J. Li , Y. Shi , C. Sun , B. An , H.‐M. Cheng , F. Li , Chem. Soc. Rev. 2021, 50, 3178. 33480899 10.1039/d0cs01017k

[184] A. A. Franco , A. Rucci , D. Brandell , C. Frayret , M. Gaberscek , P. Jankowski , P. Johansson , Chem. Rev. 2019, 119, 4569. 30859816 10.1021/acs.chemrev.8b00239 PMC6460402

[185] A. Jain , Y. Shin , K. A. Persson , Nat. Rev. Mater. 2016, 1, 15004.

[186] J. Heiska , M. Nisula , M. Karppinen , J. Mater. Chem. A 2019, 7, 18735.

[187] K. C. Kim , T. Liu , S. W. Lee , S. S. Jang , J. Am. Chem. Soc. 2016, 138, 2374. 26824616 10.1021/jacs.5b13279

[188] L. Liu , L. Miao , L. Li , F. Li , Y. Lu , Z. Shang , J. Chen , J. Phys. Chem. Lett. 2018, 9, 3573. 29897763 10.1021/acs.jpclett.8b01123

[189] Y. Zhu , K. C. Kim , S. S. Jang , J. Mater. Chem. A 2018, 6, 10111.

[190] L. Miao , L. Liu , K. Zhang , J. Chen , ChemSusChem 2020, 13, 2337. 31968154 10.1002/cssc.202000004

[191] K. H. Jung , G. S. Jeong , C. Y. Go , K. C. Kim , Energy Storage Mater. 2020, 24, 237.

[192] G. Yoon , D.‐H. Kim , I. Park , D. Chang , B. Kim , B. Lee , K. Oh , K. Kang , Adv. Funct. Mater. 2017, 27, 1702887.

[193] L. Miao , L. Liu , Z. Shang , Y. Li , Y. Lu , F. Cheng , J. Chen , Phys. Chem. Chem. Phys. 2018, 20, 13478. 29726879 10.1039/c8cp00597d

[194] M. Li , T. Liu , X. Bi , Z. Chen , K. Amine , C. Zhong , J. Lu , Chem. Soc. Rev. 2020, 49, 1688. 32101182 10.1039/c8cs00426a

[195] T. Yamashita , H. Momida , T. Oguchi , Electrochim. Acta 2016, 195, 1.

[196] Y. Chen , J. Lüder , M.‐F. Ng , M. Sullivan , S. Manzhos , Phys. Chem. Chem. Phys. 2018, 20, 232. 10.1039/c7cp06279f 29199744

[197] X. Chen , X. Liu , X. Shen , Q. Zhang , Angew. Chem. Int. Ed. 2021, 60, 24354. 10.1002/anie.202107369 34190388

[198] Y. Liu , B. Guo , X. Zou , Y. Li , S. Shi , Energy Storage Mater. 2020, 31, 434.

[199] K. Sakano , Y. Igarashi , H. Imai , S. Miyakawa , T. Saito , Y. Takayanagi , K. Nishiyama , Y. Oaki , ACS Appl. Energy Mater. 2022, 5, 2074.

[200] R. P. Carvalho , C. F. N. Marchiori , D. Brandell , C. M. Araujo , Energy Storage Mater. 2022, 44, 313.

[201] C. Lv , X. Zhou , L. Zhong , C. Yan , M. Srinivasan , Z. W. Seh , C. Liu , H. Pan , S. Li , Y. Wen , Q. Yan , Adv. Mater. 2021, 34, 2101474. 10.1002/adma.202101474 34490683

[202] S. Xu , J. Liang , Y. Yu , R. Liu , Y. Xu , X. Zhu , Y. Zhao , J. Phys. Chem. C 2021, 125, 21352.

[203] Y. Zhao , M. Wu , H. Chen , J. Zhu , J. Liu , Z. Ye , Y. Zhang , H. Zhang , Y. Ma , C. Li , Y. Chen , Nano Energy 2021, 86, 106055.

[204] K. Sakaushi , E. Hosono , G. Nickerl , T. Gemming , H. Zhou , S. Kaskel , J. Eckert , Nat. Commun. 2013, 4, 1485. 23403585 10.1038/ncomms2481

[205] A. Wild , M. Strumpf , B. Häupler , M. D. Hager , U. S. Schubert , Adv. Energy Mater. 2017, 7, 1601415.

[206] Q. Zhao , J. Wang , Y. Lu , Y. Li , G. Liang , J. Chen , Angew. Chem. Int. Ed. 2016, 55, 12528. 10.1002/anie.201607194 27608329

[207] G. Dai , Y. He , Z. Niu , P. He , C. Zhang , Y. Zhao , X. Zhang , H. Zhou , Angew. Chem. Int. Ed. 2019, 58, 9902. 10.1002/anie.201901040 30950183

[208] J. Xie , Z. Wang , Z. J. Xu , Q. Zhang , Adv. Energy Mater. 2018, 8, 1703509.

[209] Z. Tie , S. Deng , H. Cao , M. Yao , Z. Niu , J. Chen , Angew. Chem. Int. Ed. 2022, 134, e202115180. 10.1002/anie.202115180 34918433

[210] W. Yan , C. Wang , J. Tian , G. Zhu , L. Ma , Y. Wang , R. Chen , Y. Hu , L. Wang , T. Chen , J. Ma , Z. Jin , Nat. Commun. 2019, 10, 2513. 31175299 10.1038/s41467-019-10607-0 PMC6555790

[211] M. Mao , S. Wang , Z. Lin , T. Liu , Y.‐S. Hu , H. Li , X. Huang , L. Chen , L. Suo , Adv. Mater. 2021, 33, 2005781. 10.1002/adma.202005781 33470470

[212] Y. Li , Y. Lu , Y. Ni , S. Zheng , Z. Yan , K. Zhang , Q. Zhao , J. Chen , J. Am. Chem. Soc. 2022, 144, 8066. 35481353 10.1021/jacs.2c00296

[213] X. Gao , X. Zheng , Y. Tsao , P. Zhang , X. Xiao , Y. Ye , J. Li , Y. Yang , R. Xu , Z. Bao , Y. Cui , J. Am. Chem. Soc. 2021, 143, 18188. 34677957 10.1021/jacs.1c07754

[214] H. Duan , K. Li , M. Xie , J.‐M. Chen , H.‐G. Zhou , X. Wu , G.‐H. Ning , A. I. Cooper , D. Li , J. Am. Chem. Soc. 2021, 143, 19446. 34731564 10.1021/jacs.1c08675

[215] P. Peng , L. Shi , F. Huo , S. Zhang , C. Mi , Y. Cheng , Z. Xiang , ACS Nano 2019, 13, 878. 30609343 10.1021/acsnano.8b08667

[216] Y. Li , L. Liu , C. Liu , Y. Lu , R. Shi , F. Li , J. Chen , Chem 2019, 5, 2159.

[217] A. Iordache , D. Bresser , S. Solan , M. Retegan , M. Bardet , J. Skrzypski , L. Picard , L. Dubois , T. Gutel , Adv. Sustainable Syst. 2017, 1, 1600032.

[218] Z. Lin , T. Liu , X. Ai , C. Liang , Nat. Commun. 2018, 9, 5262. 30531912 10.1038/s41467-018-07599-8 PMC6288112

[219] M. H. Lee , J. Lee , S. Jung , D. Kang , M. S. Park , G. D. Cha , K. W. Cho , J. Song , S. Moon , Y. S. Yun , S. J. Kim , Y. W. Lim , D. Kim , K. Kang , Adv. Mater. 2021, 33, 2004902. 10.1002/adma.202004902 33533125

[220] T. Sun , Z. Li , H. Wang , D. Bao , F. Meng , X. Zhang , Angew. Chem. Int. Ed. 2016, 55, 10662. 10.1002/anie.201604519 27485314

[221] H. Qian , M. J. Counihan , H. A. Doan , N. A. Ibrahim , A. S. Danis , W. Setwipatanachai , N. S. Purwanto , J. Rodríguez‐López , R. S. Assary , J. S. Moore , J. Mater. Chem. A 2022, 10, 7739.

[222] H. Wang , X. Zhang , Chem. Eur. J. 2018, 24, 18235. 30007002 10.1002/chem.201802517

[223] X. Wei , W. Pan , W. Duan , A. Hollas , Z. Yang , B. Li , Z. Nie , J. Liu , D. Reed , W. Wang , V. Sprenkle , ACS Energy Lett. 2017, 2, 2187.

[224] Y. Chen , S. Zhuo , Z. Li , C. Wang , EnergyChem 2020, 2, 100030.

[225] D. Xu , M. Liang , S. Qi , W. Sun , L.‐P. Lv , F.‐H. Du , B. Wang , S. Chen , Y. Wang , Y. Yu , ACS Nano 2021, 15, 47. 33382596 10.1021/acsnano.0c05896

[226] P. Poizot , Y. Yao , J. Chen , U. S. Schubert , ChemSusChem 2020, 13, 2107. 32347656 10.1002/cssc.202001022

[227] L. Zhou , S. Jo , M. Park , L. Fang , K. Zhang , Y. Fan , Z. Hao , Y. Kang , Adv. Energy Mater. 2021, 11, 2003054.

[228] Y. Sun , S. Guo , H. Zhou , Adv. Energy Mater. 2019, 9, 1800212.

[229] L. Zhao , A. E. Lakraychi , Z. Chen , Y. Liang , Y. Yao , ACS Energy Lett. 2021, 6, 3287.

